# Electrolyte and metabolite composition of cystic fluid from a rat model of ARPKD

**DOI:** 10.1038/s42003-025-07631-w

**Published:** 2025-02-13

**Authors:** Christine A. Klemens, Mykhailo Fedoriuk, Marharyta Semenikhina, Mariia Stefanenko, Adrian Zietara, Vladislav Levchenko, Lashodya V. Dissanayake, Oleg Palygin, Alexander Staruschenko

**Affiliations:** 1 Department of Molecular Pharmacology and Physiology, University of South Florida, Tampa, FL USA; 2 Hypertension and Kidney Research Center, University of South Florida, Tampa, FL USA; 3 Department of Medicine, Medical University of South Carolina, Charleston, SC USA; 4 James A. Haley Veterans’ Hospital, Tampa, FL USA

**Keywords:** Kidney, Metabolism, Polycystic kidney disease

## Abstract

Fluid-filled cysts are the key feature of polycystic kidney disease, which eventually leads to renal failure. We analyzed the composition of cyst fluid from a rat model of autosomal recessive polycystic kidney disease, the PCK rat, and identified sexual differences. Our results demonstrate that the ion composition of cyst fluid differs from that of urine or plasma. Untargeted metabolomics combined with transcriptomic data identified tryptophan metabolism, enzyme metabolism, steroid hormone biosynthesis, and fatty acid metabolism as pathways differing between male and female PCK rats. We quantified 42 amino acids in the cyst fluid (PCK only), plasma, and urine of male and female PCK rats and Sprague Dawley rats. Taurine was the most concentrated amino acid present in the cyst fluid, and PCK rat urinary taurine excretion was over 3-fold greater than Sprague Dawley rats. Understanding the composition of cyst fluid provides valuable insights into disease pathophysiology and may help identify potential dietary or pharmacological interventions to mitigate disease progression and improve patient outcomes.

## Abstract (web summary)

Untargeted metabolomics and amino acid analysis of cystic fluid in a polycystic kidney disease (PKD) rat model reveal novel insights into sex differences and the role of taurine in PKD progression.

## Introduction

Polycystic Kidney Disease (PKD) is a devastating inherited kidney disease that is a significant cause of kidney failure. Large fluid-filled cysts develop in the kidney and continually expand over time, damaging the surrounding tissue until renal replacement therapy is required. PKD has both autosomal-dominant (ADPKD) and autosomal-recessive (ARPKD) forms. ADPKD is caused predominantly by mutations in *PKD1* and *PKD2*, and typically manifests in adulthood^1^. Most studies have reported that male sex is associated with a faster disease progression, but the reason behind this is not known^2–5^. In contrast, ARPKD is much less common; however, the disease progression is more severe and manifests perinatally or in childhood^6,7^. ARPKD is caused by a mutation in *PKHD1*, which encodes fibrocystin (also known as polyductin)^8^. Despite the severity of the disease, to date there is only one treatment option, Tolvaptan, available for ADPKD^9^, and none for ARPKD (although the same drug is currently in clinical trials^10^). Tolvaptan is a vasopressin V2 receptor antagonist with unpleasant adverse effects, including polyuria, polydipsia, and potential hepatotoxicity^11^.

Because the development of cysts is the major feature of this disease, researchers have long sought to determine the composition of cystic fluid. Foundational studies by Bricker and Patton^12^, Gardner^13,14^, Grantham^15,16^, and Huseman et al.^17^, observed that in ADPKD patients, cysts have heterogenous and variable ion concentrations. In general, cysts more associated with the proximal nephron segments had ion concentrations similar to concentrations in the interstitial space (high Na^+^, high Cl^−^, low K^+^). Interestingly, more distal cysts had an inverse ion balance, with low Na^+^ (2–50 mM), low Cl^−^ (0–55 mM), and high K^+^ (5–58 mM)^13–16^. The osmolality of both proximal and distal cysts was reported to be around 300 mOsm; however, osmolar calculations of the distal cysts had an osmolar gap, 50–100 mOsm of which was likely compensated for by amino acids^13,14,17^. An additional study from Rohatgi et al. found that ion concentrations from 3 ARPKD patients had Na^+^, K^+^ and osmolality concentrations similar to the distal cysts in the ADPKD patients^18^. Lastly, the ion concentrations and osmolality of cyst fluid from Madin Darby Canine Kidney cell in vitro cysts, were 159 ± 7 mM Na^+^, 4.7 ± 0.1 mM K^+^, and 138 ± 6 mM Cl^−^^19^. To the best of our knowledge, no one has previously published the ion concentrations of cystic fluid from any PKD rodent models.

In addition to the earlier studies, there have been a few studies that used nuclear magnetic resonance (NMR) spectroscopy or proteomics to further interrogate the cyst fluid milieu^20–22^. The NMR study from Foxall et al. found both large macromolecules as well as low-molecular-weight compounds, including amino acids, sugars, polyols, and organic acids and bases^20^. Remarkably, this study found little variation in the NMR spectra from different patients, and several amino acids had concentrations substantially greater than their plasma or urinary values, including leucine, isoleucine, and lysine^20^. A study seeking to characterize the ADPKD kidney cyst fluid proteome identified over 350 unique proteins, including a preponderance of Ig and related immune proteins, a large number of SERPIN family proteins, and vitronectin and clusterin, among others^21^. There was an enrichment of stress and wounding response, immune and inflammatory response, complement activation, and regulation of body fluids protein categories^21^.

With the emergence of the omics era, a number of studies have interrogated the metabolomic composition of plasma, urine, and kidney tissue from PKD patients or rodent models of PKD^23–32^. These studies have had a range of goals, including looking for biomarkers to trace disease severity and progression, to identify pathophysiological metabolic derangements that occur during disease progression, and find novel dysregulated metabolic pathways that could be targeted for therapeutic intervention. As transport physiologists, our approach to thinking about cyst fluid composition and rationale for performing metabolomics analysis was a bit different. If certain compounds are undergoing trans- or paracellular transport into the cyst and accumulating there, this could exacerbate cystogenesis. Additionally, since many transporters rely on Na^+^ gradients for secondary active transport, knowing the ion concentrations of the cystic fluid in relation to the other solutes and proteins increases our understanding of how certain solutes might get trapped.

The overall goal of this study was to characterize the cyst fluid composition of a rat model of ARPKD, the PCK rat. This model developed spontaneously and features a mutation in *Pkhd1*, the ortholog to the causative ARPKD gene in humans^33,34^. The PCK rat develops both renal and hepatic cysts, and males have exacerbated renal damage starting around 18 weeks of age compared to females^33,35^. Also, similar to ARPKD patients, the cysts arise predominantly from the collecting duct^35^. The rationale for using this model was to eliminate potential variation from a heterogeneous cyst population that might occur in an ADPKD model. An additional reason is because in humans, a considerable portion of the cysts are believed to arise more frequently from the distal tubule and collecting duct, and these tight epithelia are likely to rely on transcellular transport mechanisms^36^. Beyond this, we wanted to compare cyst fluid composition between males and females at a point prior to males having significantly worse kidney-to-body weight ratios to identify compounds or metabolic pathways that may be deleterious in males or protective in females. Investigation into cyst fluid composition not only advances our comprehension of PKD pathology but also highlights the potential of metabolomics in identifying novel biomarkers for disease progression and uncovering pathophysiological processes regulating transport mechanisms in cyst formation.

## Results

### Cyst fluid from PCK rats has a distinct ionic composition

**Fig. 1:**
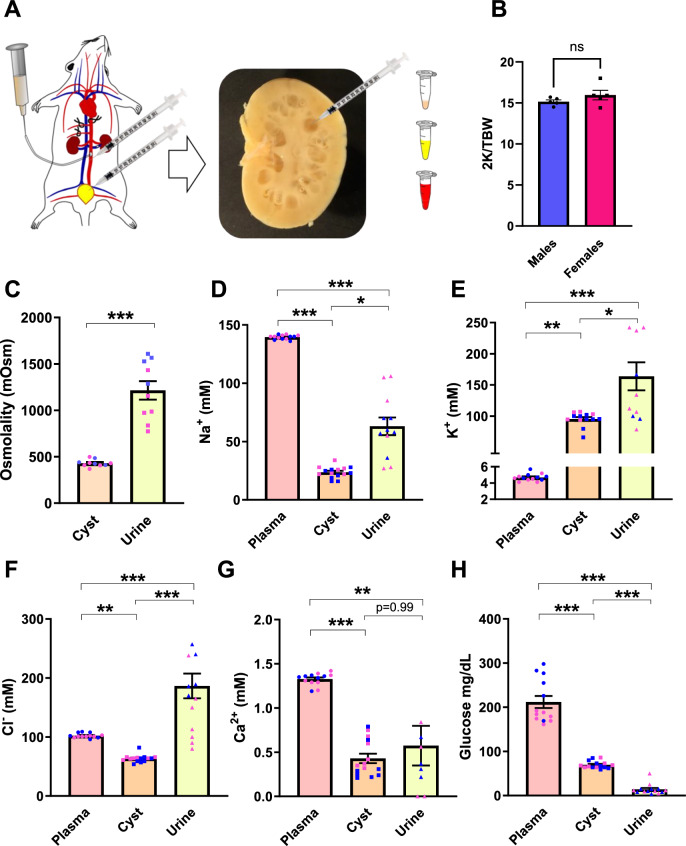
Cyst fluid collection and electrolyte composition. **A** Male and Female (10–12 weeks-old) PCK rat kidneys were flushed with PBS, sectioned, and cyst fluid was immediately carefully aspirated from intact cysts and snap-frozen. Blood from the abdominal aorta and urine directly from the bladder were also collected. **B** There is no significant difference in 2 kidney/total body weight ratios (2 K/TBW) at the collected age. **C** Cyst and Urine osmolalities (**D**) Na^+^ concentrations (**E**) K^+^ concentrations (**F**) Cl^−^ concentrations (**G**) Ca^2+^ concentrations (**H**) Non-fasted glucose concentrations. Individual male (blue) and female (pink) data points are shown in each graph. Significance was determined by unpaired *t*-test (2 K/TBW, Osmolality) or Brown-Forsythe and Welch ANOVA with Dunnet’s correction *p* < 0.05 considered significant. *N* ≥ 5 rats. Graphs demonstrate the mean ± SEM. *p < 0.05 **p < 0.01 ***p < 0.001.

For these experiments, blood was collected from the abdominal aorta, urine was collected directly from the bladder, and cystic fluid was collected via micropuncture using an U100 syringe from male and female PBS-perfused cystic kidneys (Fig. 1A). Cyst fluid, urine, and plasma were immediately snap frozen after collection. Both males and females were studied to examine potential sexual dimorphism. At 10–12 weeks of age, there was no significant difference in the 2 kidney to body weight ratios (Fig. 1B) between male and female PCK rats. Additionally, there were no significant differences in cyst fluid or plasma solute concentrations or osmolality between males and females. The osmolality of the cystic fluid was significantly reduced compared to urine (431 ± 12 vs 1215 ± 99 mOsm, *p*-value < 0.0001, Fig. 1C), and was relatively close to what has been reported for human cystic fluid^13,15,17^. Cystic fluid values were pointedly lower than plasma or urinary values for Na^+^ (139.5 ± 0.5 vs 23.7 ± 1.2 vs 63.1 ± 7.6 mM, plasma vs cyst vs urine, Fig. 1D) and Cl^−^ (102.3 ± 1.1 vs 65.5 ± 2.1 vs 186 ± 2.0 mM, Fig. 1F). K^+^ was dramatically increased in the cystic fluid compared to plasma, but lower than urinary values (4.7 ± 0.1 vs 95.2 ± 3.1 vs 163.8 ± 22.5 mM, Fig. 1E), and Ca^2+^ was reduced compared to plasma, but not different from urine (1.32 ± 0.02 vs 0.41 ± 0.05 vs 0.37 ± 0.11 mM, Fig. 1G). Interestingly, glucose was significantly lower than plasma values (unfasted) but greater than urinary glucose (211 ± 13.7 vs 69.9 ± 2.1 vs 14.1 ± 3.1 mg/dL, Fig. 1H). Creatinine values were also greater than plasma but less than urine (Fig. S1).

### Metabolomic analysis of male and female PCK rat cystic fluid

**Fig. 2:**
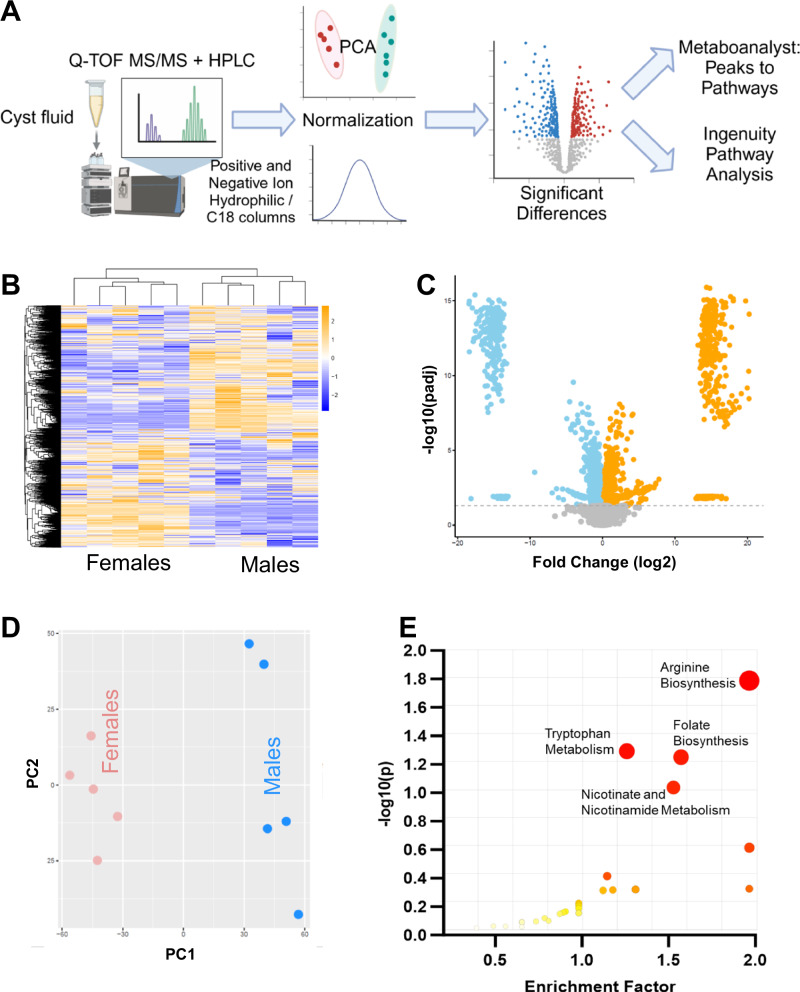
Untargeted metabolomics of male and female cyst fluid. **A** Experimental Approach. **B** Heatmap showing fold change differences for individual animals from the -C18 column results. **C** Volcano plot showing compounds that were significantly increased (orange) or decreased (blue) in females with a log2fold change (FC) > 1 with *p*-adjusted value < 0.05 as determined by differential expression analysis *R*-package DEseq2. **D** Principal Component Analysis (PCA) plot multivariate analysis showing clustering of male and female results. **E** Enrichment analysis of cyst fluid metabolic pathways that were significantly different between groups. *N* = 6 male and 6 female rats.

Figure 2A shows the experimental approach for untargeted metabolomics of the cystic fluid. Briefly, following Quadrupole Time-of-Flight Mass Spectrometry and High Performance Liquid Chromatography, compounds were identified based on their mass and charge. For further analyses, the values were normalized and assessed for grouping with principal component analysis (PCA) and statistical differences between males and females for each identified compound. In the initial identification, 6910 mass peaks were found in all 4 conditions (negative or positive ionization, C18 or hydrophobic columns). The initial data, including putative compound ID before mummichog algorithm analysis, fold change differences, p-values, peak area, mass, and retention time, can be found in the Supplemental Data Excel file. Figure 2B is a heat map showing the fold changes for individual samples that were different between males and females. The PCA plot clearly demonstrates that males and females segregate into distinct groups with intergroup similarities (Fig. 2D). As would be expected based on the PCA plots, there were a large number of peaks that were significantly up (1305, orange) or down (1214, blue) regulated in females compared to males (Fig. 2C). Additionally, there were 253 compounds in females that were absent in males, and 210 compounds found in cystic fluid from males, but not female. Lastly, the assessment of key metabolic pathways that differed between males and females was determined using Metaboanalyst Peaks to Pathways 6.0. These results are graphed in Fig. 2E. The top pathways predicted to be significantly different between males and females were arginine biosynthesis, tryptophan metabolism, folate biosynthesis, and nicotinate and nicotinamide metabolism. Beyond this, grouping chemicals by class revealed that the majority of ID’d metabolites were amino acids or peptide fragments.

### Transcriptomic analysis of male and female PCK kidney tissue

**Fig. 3:**
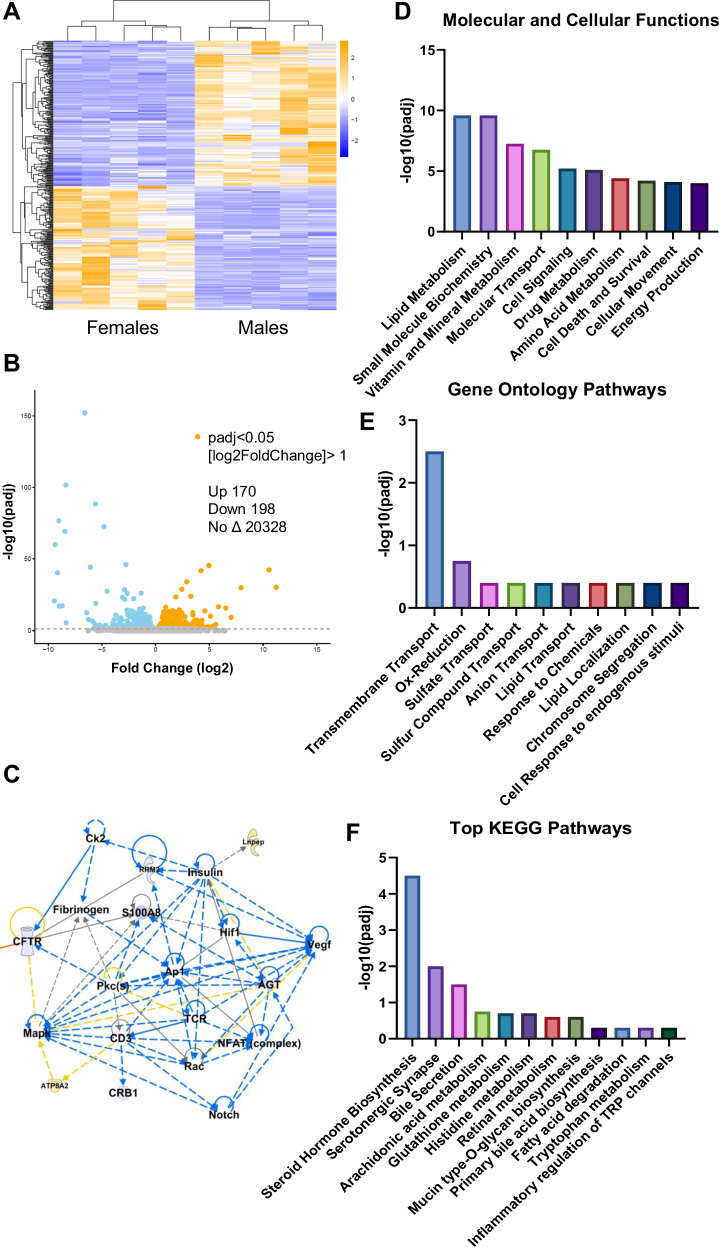
RNA-seq analysis of male and female PCK rat kidney. **A** Heatmap showing fold change differences for different animals. **B** Volcano plot of significantly up (orange) or down (blue) differentially expressed genes in females with a log2fold change (FC) > 1 and *p*-adjusted value < 0.05 as determined by differential expression analysis R-package DEseq2. **C** Signaling network demonstrating down-regulation of pathway-related genes including *Vegf*, *Notch*, *Hif1a*, and *Cftr* in females. Top Molecular and Cellular Functions (**D**), Gene Ontology Pathways (**E**), and KEGG Pathways (**F**) that are predicted to be different between male and female PCK kidney. *N* = 5 male and 5 female rats.

To further solidify sex differences contributing to the distinct male and female cystic profiles, we performed RNA-Seq analysis of renal cortex tissue from male and female PCK rats used for untargeted metabolomics. The full RNA-Seq dataset can be found in the Supplemental Data. The heatmap in Fig. 3A depicts the top up- and downregulated genes in females compared to males. After considering an adjusted *p*-value cutoff of 0.05 and log2 fold-change cutoff > 1, we found that 170 genes were upregulated and 198 were downregulated in females (Fig. 3B). Ingenuity Pathway Analysis (IPA) of this dataset revealed the top molecular and cellular functions associated with the differentially expressed genes (Fig. 3D), the top Gene Ontology (GO, Fig. 3E), and top Kyoto-Encyclopedia of Genes and Genomes (KEGG, Fig. 3F) pathways. Lastly, IPA identified a key signaling network involving the cystic fibrosis transmembrane regulator (*CFTR*), *Notch*, *Rac, Vegf*, *S100A8*, *Hif1*, and others whose expression and downstream activities are predicted to be less active (blue) in females (Fig. 3C). Increased circulating levels of VEGF^37^ and HIF-1a^38^ are associated with worsened PKD disease state, S100A8 upregulation is linked to receptor of advanced glycation end product (RAGE) signaling in inflammation and cystogenesis^39^, overexpression of Notch1 in renin-expressing cells resulted in development of fluid-filled cysts and increased proliferation markers^40^, and CFTR is a key chloride channel that may contribute to cyst growth^41^.

### Comparison of key ion channels in the collecting duct

**Fig. 4:**
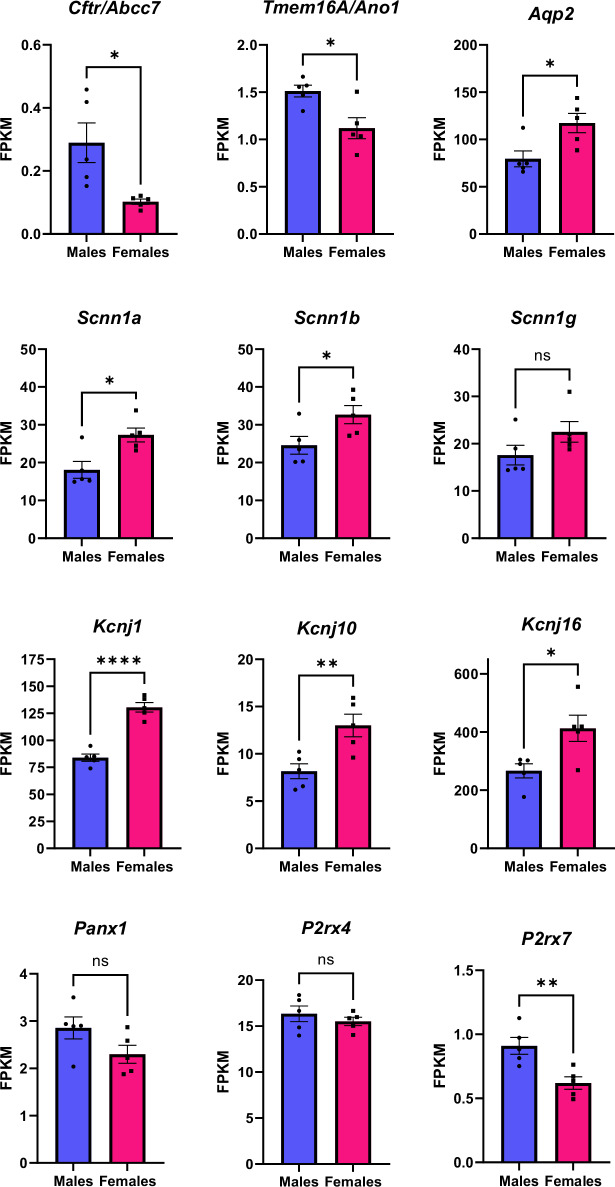
Differentially expressed ion channel genes expressed in the collecting duct. *Cftr* and *Tmem16A* encode chloride channels: cystic fibrosis transmembrane regulator (CFTR) and the calcium-activated chloride channel Anoctamin. *Aqp2* is the gene that transcribed aquaporin 2. Scnn1a, Scnn1b, and Scnn1g encode the α, β, and γ subunits of the epithelial Na^+^ channel (ENaC), respectively. *Kcnj1*, *Kcnj10*, and *Kcnj16* encode the inward-rectifying K^+^ channels: ROMK, K_ir_4.1 and K_ir_5.1. *Panx1*, *P2rx4*, and *P2rx7* transcribe an ATP-releasing channel (Pannexin1) and Ca^+^ transporting ionic purinergic receptors P2X4 and P2X7. Significance was determined by unpaired two-tailed *t*-test with *p* < 0.05 considered significant. Graphs demonstrate the mean ± SEM. *p < 0.05 **p < 0.01 ***p < 0.001. KPKM - Fragments Per Kilobase per Million mapped fragments. *N* = 5 male and 5 female rats.

It is well established that ion channels and transporters along the nephron display strong sexual dimorphism^42,43^. Additionally, there are a number of key channels whose activity may contribute to cystogenesis, including the Epithelial Na^+^ Channel (ENaC)^44^, Aquaporin 2 (AQP2), CFTR, TMEM16A (also known as anoctamin), and Pannexin 1^41^. While we did not see any significant differences in the Na^+^, Ca^2+^, K^+^, or Cl^−^ concentrations in the cystic fluid, we assessed normalized gene counts (Fragments Per Kilobase of transcript per Million mapped reads, FPKM) of ion channels associated with cyst growth in the collecting duct. Many key channel transcripts were significantly different between males and females, including α- and β-ENaC subunits (α *Scnn1a* and β *Scnn1b*), ROMK (*Kcnj1*), K_ir_4.1 (*Kcnj10*), K_ir_5.1 (*Kcnj16*), *Aqp2*, *Cftr*, *Tmem16A*, and the ionic purinergic receptor, *P2rx7* (Fig. 4). To estimate correlation of RNA-seq data with protein levels, we have assessed protein levels of K_ir_5.1 with Western blotting. We found a significant increase in K_ir_5.1 protein in the PCK females consistent with the transcript expression levels (Fig. S2). Changes in the protein levels for several other channels and transporters have been thoroughly analyzed and discussed in the discussion section.

### Joint Pathway analysis of metabolomic and transcriptomic data

**Fig. 5:**
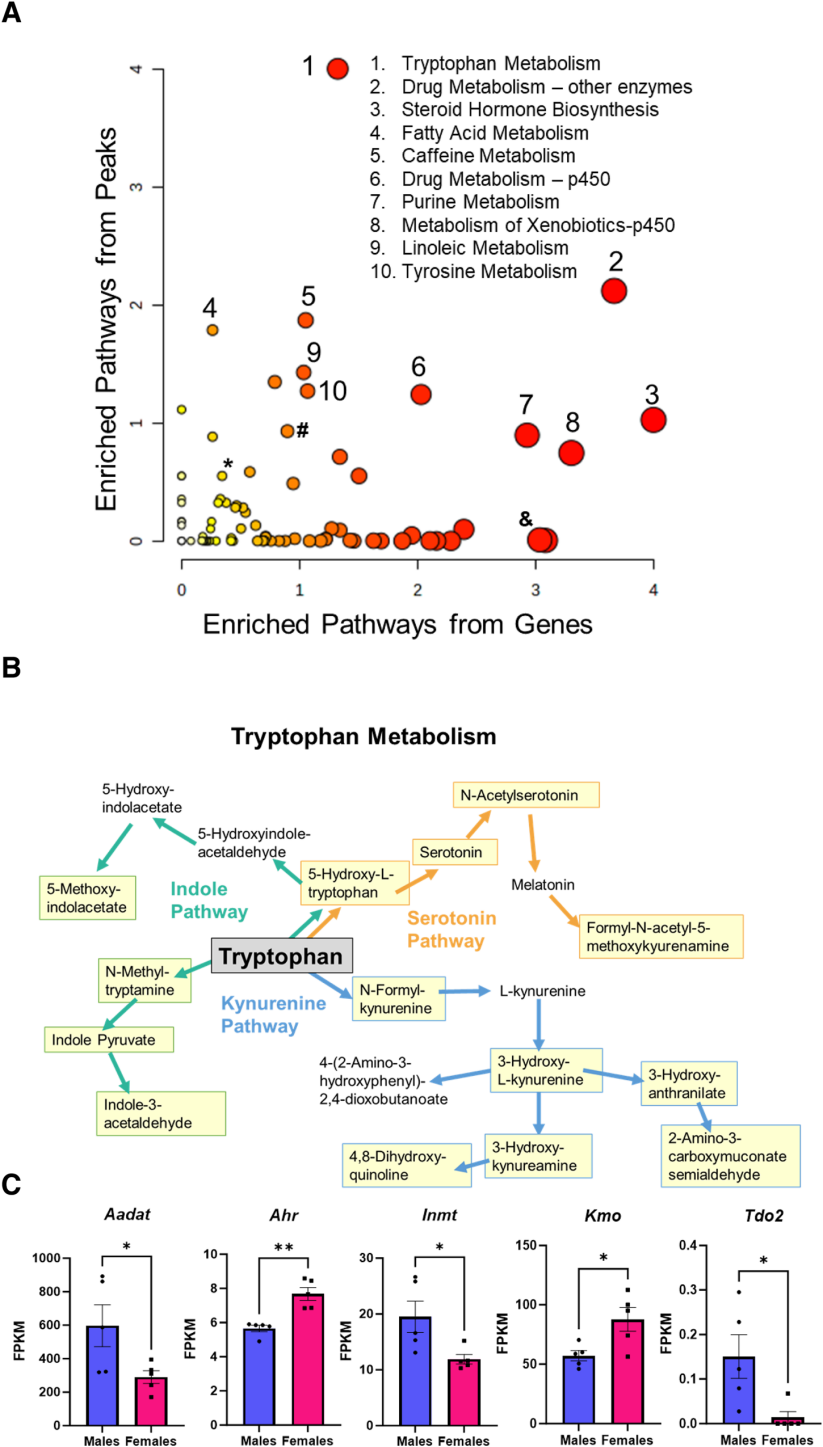
Joint pathway analysis of metabolomics dataset with RNAseq dataset. **A** Enrichment analysis of metabolic pathways resulting from the identification of significantly different cyst fluid metabolites paired with differentially expressed genes in male and female PCK rat kidneys. Larger *y*-axis coordinates correspond to a greater number of significantly different metabolites from a pathway. A larger *x*-axis coordinates result from increased numbers of significantly different genes in a pathway. Major pathways predicted to be different after combining both metabolite and gene expression include: tryptophan metabolism, drug metabolism, steroid hormone biosynthesis, fatty acid metabolism, caffeine metabolism, drug metabolism by cytochrome p450, purine metabolism, metabolism of xenobiotics by cytochrome p450, linoleic metabolism, and tyrosine metabolism. Also, of note are # - amino sugar and nucleotide sugar metabolism, & - arginine and proline metabolism, and * - taurine and hypotaurine metabolism. **B** Schematic of the 3 major arms of tryptophan metabolism. Compounds in yellow boxes are significantly different between male and female cyst fluid. **C** Graphs showing significant differences in several genes associated with tryptophan metabolism signaling. Graphs demonstrate the mean ± SEM. Significance was determined by unpaired two-tailed *t*-test *p < 0.05 **p < 0.01 ***p < 0.001. KPKM - Fragments Per Kilobase per Million mapped fragments.

Following the RNA-Seq analysis, we performed a joint pathway analysis by overlaying metabolite changes with transcript changes in Metaboanalyst 6.0 to further delineate metabolic pathways that are different between male and female PCK rats. Figure 5A shows the enriched pathway plot, where greater fold-change differences in genes are graphed on the *x*-axis and greater fold-changes in predicted metabolites are graphed as y-coordinates. The full analysis table for this comparison can be found in the Supplemental Data. Tryptophan metabolism remained a top hit, along with drug metabolism, steroid hormone biosynthesis, and fatty acid metabolism. Tryptophan metabolism occurs through 3 branches: the indole pathway, the serotonin pathway, and the kynurenine pathway. Several compounds significantly differed between males and females in all 3 branches. Figure 5B demonstrates these branches, and all compounds in yellow boxes are different in males and females. To continue looking at this pathway in more detail, we assessed the FPKM values of several genes involved in tryptophan metabolism and found significant differences in *Aadat*, *Ahr*, *Inmt*, *Kmo*, and *Tdo2* expression (Fig. 5C). No differences were observed for *Ido1*, *Ido2*, or *Kynu* (data not shown, but can be found in RNA-Seq supplementary materials).

### Quantification of amino acid concentrations in cyst fluid

**Fig. 6:**
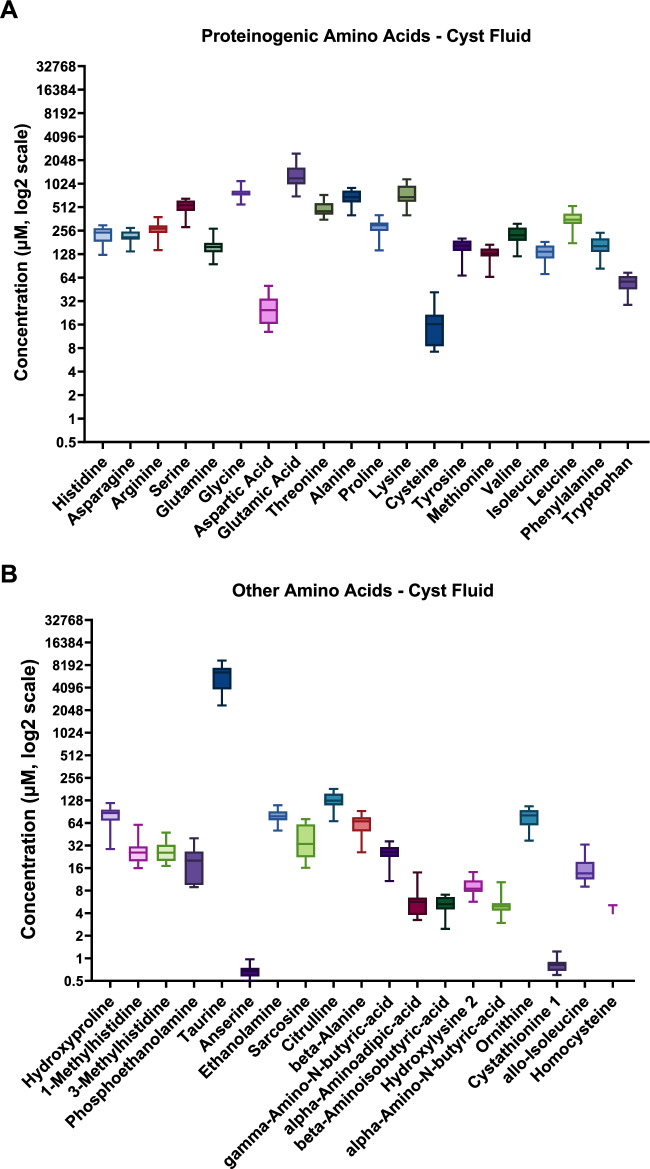
Targeted metabolomic assay to quantify amino acid concentrations present in cystic fluid. **A** Quantification of amino acids used as building blocks for proteins. **B** Quantification of non-proteinogenic amino acids. Box plots demonstrate the mean amino acid concentrations. Both male and female values are included in the average (*N* = 12). Values were determined via mass spectroscopy of cyst fluid compared against known standards. Taurine is the most abundant amino acid present in cystic fluid. Graph *y*-axis are the same scale for easy comparison of relative abundance. *N* = 6 male and 6 female rats.

We directly quantified the concentrations of amino acids present in the cystic fluid using a targeted metabolomics approach. We also quantified the plasma and urine values for both PCK and SD control rats for comparison. All cyst fluid, plasma, and urine amino acid concentrations and pairwise comparisons can be found in Tables 1–3. Please note that the SD and PCK rats were kept on slightly different diets and collected at different institutes, so values within normal ranges that are significantly different between the two rats’ strains should be approached with caution. Figure 6 shows the relative abundance of the 42 amino acids we tested in cyst fluid. The top 5 most plentiful amino acids in descending order are: Taurine, Glutamic Acid, Glycine, Alanine, and Lysine. While Tryptophan was consistently observed in the omics analyses, its abundance was lower (56.6 ± 3.9 µM) than many other amino acids. Mean male and female values are shown in Table 1.

**Table 1:** Cyst fluid amino acid concentrations

Cyst fluid amino acid (µM)	PCK Rats		
	Males	Females	
	Mean ± SEM	Mean ± SEM	
Histidine	236.58 ± 18.51	219.97 ± 26.27	
Hydroxyproline	87.88 ± 4.40	74.62 ± 13.11	
1-Methylhistidine	20.68 ± 1.56	35.13 ± 5.39	#
3-Methylhistidine	20.93 ± 1.57	33.68 ± 3.40	#
Asparagine	212.12 ± 9.03	220.78 ± 20.27	
Phosphoethanolamine	21.38 ± 4.58	20.28 ± 4.68	
Arginine	268.88 ± 9.33	280.20 ± 36.35	
Carnosine	0.00 ± 0.00	0.00 ± 0.00	
Taurine	7508.12 ± 469.11	4556.87 ± 788.33	#
Anserine	0.70 ± 0.05	0.67 ± 0.07	
Serine	532.83 ± 30.78	524.37 ± 55.78	
Glutamine	161.82 ± 24.05	161.42 ± 10.34	
Ethanolamine	91.63 ± 5.31	69.68 ± 4.84	#
Glycine	829.73 ± 56.68	717.23 ± 45.50	
Aspartic Acid	22.38 ± 3.25	30.97 ± 5.55	
Sarcosine	27.80 ± 3.32	49.48 ± 9.44	
Citrulline	125.15 ± 8.26	135.35 ± 16.21	
Glutamic Acid	1317.40 ± 249.57	1365.98 ± 152.75	
Beta-Alanine	73.10 ± 4.23	55.43 ± 8.55	
Threonine	455.42 ± 33.11	535.18 ± 58.85	
Alanine	685.07 ± 52.34	717.87 ± 71.65	
Gamma-Amino-N-butyric-acid	28.60 ± 2.17	23.00 ± 2.77	
Alpha-Aminoadipic-acid	5.91 ± 1.66	5.89 ± 0.55	
Beta-Aminoisobutyric-acid	5.47 ± 0.49	5.12 ± 0.65	
Proline	279.83 ± 13.12	307.03 ± 37.06	
Hydroxylysine 1	0.00 ± 0.00	0.00 ± 0.00	
Hydroxylysine 2	8.04 ± 0.19	10.50 ± 1.16	0.06
Alpha-Amino-N-butyric-acid	5.77 ± 0.94	4.68 ± 0.40	
Ornithine	85.39 ± 5.44	70.03 ± 10.16	
Cystathionine 1	0.85 ± 0.06	0.82 ± 0.10	
Cystathionine 2	0.00 ± 0.00	0.00 ± 0.00	
Lysine	627.73 ± 31.61	853.52 ± 111.47	
Cysteine	18.57 ± 4.96	17.78 ± 3.97	
Tyrosine	170.43 ± 8.49	146.40 ± 20.21	
Methionine	127.27 ± 4.98	134.62 ± 15.62	
Valine	227.42 ± 13.46	233.42 ± 30.31	
Isoleucine	134.47 ± 8.23	138.33 ± 18.55	
Allo-Isoleucine	13.58 ± 1.48	17.44 ± 3.48	
Homocysteine	2.07 ± 0.96	0.97 ± 0.72	
Leucine	358.57 ± 17.54	362.15 ± 52.84	
Phenylalanine	164.05 ± 9.53	169.82 ± 24.12	
Tryptophan	60.78 ± 4.14	52.43 ± 6.52	

# Significantly different between Male and Female PCK Rats.

Pairwise comparisons of cystic fluid were conducted using Mann–Whitney *U* tests (non-parametric data), # *p*-value < 0.05, *N* = 6 rats per group.

**Table 2:** Plasma amino acid concentrations

Plasma amino acid (µM)	Sprague Dawley		PCK Rats		Comparison			
	Males	Females	Males	Females	SD	PCK	M	F
	Mean ± SEM	Mean ± SEM	Mean ± SEM	Mean ± SEM	F vs M	F vs M	PCK vs SD	PCK vs SD
Histidine	64.22 ± 1.39	52.47 ± 2.11	99.78 ± 11.72	87.50 ± 6.63	*		0.06	§
Hydroxyproline	49.17 ± 2.05	25.98 ± 1.06	82.10 ± 6.94	38.28 ± 4.66	*	#	¥	0.06
1-Methylhistidine	19.77 ± 0.98	12.65 ± 0.23	13.42 ± 1.11	9.07 ± 0.50	*	#	¥	§
3-Methylhistidine	12.27 ± 0.91	7.23 ± 0.20	6.62 ± 0.56	4.68 ± 0.53	*	#	¥	§
Asparagine	102.37 ± 3.95	79.80 ± 2.64	104.42 ± 4.82	86.45 ± 8.06	*			
Phosphoethanolamine	5.43 ± 0.24	4.83 ± 0.37	19.40 ± 2.67	13.20 ± 1.79			¥	§
Arginine	134.20 ± 3.72	123.57 ± 4.56	107.52 ± 4.31	115.88 ± 3.70			¥	
Carnosine	2.13 ± 0.21	1.18 ± 0.13	0.00 ± 0.00	0.00 ± 0.00	*		¥	§
Taurine	202.37 ± 16.79	194.45 ± 6.96	164.87 ± 8.75	128.37 ± 3.90		#	¥	§
Anserine	4.48 ± 0.37	3.98 ± 0.31	1.08 ± 0.11	1.19 ± 0.19			¥	§
Serine	322.48 ± 17.58	263.83 ± 10.10	326.00 ± 13.73	258.05 ± 14.60	*	#		
Glutamine	487.52 ± 3.44	396.55 ± 14.24	559.82 ± 24.24	479.82 ± 16.97	*	#	0.06	§
Ethanolamine	8.62 ± 0.26	7.03 ± 0.49	7.57 ± 0.42	5.13 ± 0.29	*	#	¥	§
Glycine	394.53 ± 20.90	229.18 ± 5.91	151.33 ± 6.56	99.38 ± 8.45	*	#	¥	§
Aspartic Acid	2.60 ± 0.18	1.93 ± 0.18	2.58 ± 0.51	2.32 ± 0.25	*			
Sarcosine	2.30 ± 0.07	1.52 ± 0.12	1.45 ± 0.11	0.93 ± 0.17	*	#	¥	§
Citrulline	66.88 ± 1.17	73.28 ± 3.76	72.02 ± 2.48	84.43 ± 2.00		#		§
Glutamic Acid	72.17 ± 5.19	48.38 ± 2.61	77.63 ± 11.53	62.47 ± 8.39	*			
Beta-Alanine	1.23 ± 0.10	0.85 ± 0.11	1.18 ± 0.22	0.78 ± 0.17	*			
Threonine	301.55 ± 11.69	313.33 ± 19.21	349.20 ± 23.42	397.68 ± 29.62			¥	
Alanine	471.12 ± 35.13	458.85 ± 33.07	559.90 ± 23.67	576.25 ± 40.74				
Gamma-Amino-N-butyric-acid	0.01 ± 0.00	0.00 ± 0.00	0.11 ± 0.02	0.08 ± 0.01			¥	§
Alpha-Aminoadipic-acid	2.25 ± 0.08	1.82 ± 0.10	1.68 ± 0.15	1.52 ± 0.16	*		¥	
Beta-Aminoisobutyric-acid	0.10 ± 0.00	0.10 ± 0.00	0.09 ± 0.00	0.09 ± 0.00			¥	§
Proline	157.73 ± 5.97	168.92 ± 5.85	254.70 ± 22.17	250.23 ± 9.97			¥	§
Hydroxylysine 1	0.10 ± 0.00	0.10 ± 0.00	0.00 ± 0.00	0.00 ± 0.00			¥	§
Hydroxylysine 2	1.99 ± 0.18	1.70 ± 0.14	0.65 ± 0.11	0.68 ± 0.07			¥	§
Alpha-Amino-N-butyric-acid	18.42 ± 1.48	43.02 ± 4.60	5.44 ± 0.24	5.40 ± 0.43	*		¥	§
Ornithine	51.38 ± 2.30	27.95 ± 2.44	63.04 ± 5.48	38.56 ± 1.81	*	#		§
Cystathionine 1	1.02 ± 0.09	0.43 ± 0.03	0.74 ± 0.05	0.72 ± 0.03	*		¥	§
Cystathionine 2	0.00 ± 0.00	0.00 ± 0.00	0.00 ± 0.00	0.00 ± 0.00				
Lysine	504.40 ± 22.20	486.43 ± 20.00	297.28 ± 10.15	404.27 ± 15.24		#	¥	§
Cysteine	38.18 ± 0.47	45.47 ± 2.84	29.47 ± 1.76	32.93 ± 2.25	0.06		¥	§
Tyrosine	88.88 ± 3.86	62.88 ± 7.01	70.83 ± 4.20	46.10 ± 2.86	*	#	¥	0.06
Methionine	61.25 ± 1.18	55.92 ± 1.32	64.73 ± 4.80	56.15 ± 2.44	*			
Valine	232.58 ± 7.15	163.40 ± 9.53	123.08 ± 8.43	124.52 ± 9.66	*		¥	§
Isoleucine	132.87 ± 3.62	79.78 ± 5.12	64.10 ± 5.80	61.25 ± 5.92	*		¥	0.06
Allo-Isoleucine	0.00 ± 0.00	0.23 ± 0.11	0.33 ± 0.05	0.46 ± 0.05	0.06		¥	
Homocysteine	0.83 ± 0.09	0.47 ± 0.07	0.00 ± 0.00	1.67 ± 1.55	*		¥	
Leucine	197.43 ± 4.74	131.67 ± 6.55	168.75 ± 10.19	156.02 ± 13.11	*		0.06	
Phenylalanine	64.73 ± 1.22	58.37 ± 1.96	58.12 ± 3.99	50.08 ± 1.86	*		0.06	§
Tryptophan	125.57 ± 1.95	139.05 ± 4.50	59.13 ± 2.92	65.17 ± 4.38	*		¥	§

Pairwise comparisons of cystic fluid were conducted using Mann–Whitney *U* tests (non-parametric data), *p*-value < 0.05 are considered significant. *N* = 6 rats per group.

* Significantly different between Male and Female SD rats

# Significanly different betweem Male and Female PCK Rats

¥ Significantly different between Male SD and PCK rats

§ Significantly different between Female SD and PCK rats

**Table 3:** Urine fluid amino acid concentrations

Urine amino acid (µM)	Sprague Dawley		PCK Rats		Comparison			
	Males	Females	Males	Females	SD	PCK	M	F
	Mean ± SEM	Mean ± SEM	Mean ± SEM	Mean ± SEM	FvM	FvM	PCKvSD	PCKvSD
Histidine	24.85 ± 7.96	33.42 ± 16.35	169.30 ± 65.12	49.42 ± 14.78			¥	
Hydroxyproline	32.38 ± 13.57	27.53 ± 19.30	34.63 ± 7.80	14.12 ± 3.97		#		
1-Methylhistidine	17.45 ± 6.00	143.05 ± 89.12	11.50 ± 2.16	32.77 ± 17.70	*			0.06
3-Methylhistidine	8.03 ± 1.94	33.38 ± 8.15	8.57 ± 2.25	25.00 ± 9.58	*			
Asparagine	29.62 ± 9.30	29.90 ± 12.36	173.78 ± 56.80	59.98 ± 16.37			¥	0.06
Phosphoethanolamine	1.03 ± 0.07	0.80 ± 0.03	4.07 ± 1.45	1.57 ± 0.56	*		¥	
Arginine	21.87 ± 5.19	16.15 ± 4.68	129.07 ± 51.35	30.20 ± 7.91			¥	
Carnosine	0.00 ± 0.00	0.00 ± 0.00	0.00 ± 0.00	0.00 ± 0.00				
Taurine	2043.65 ± 613.67	2394.68 ± 751.55	9704.97 ± 1863.02	7569.93 ± 1630.58			¥	§
Anserine	0.32 ± 0.09	0.35 ± 0.11	0.63 ± 0.11	0.47 ± 0.05				
Serine	59.58 ± 15.58	54.77 ± 20.32	413.53 ± 170.45	68.02 ± 16.30			¥	
Glutamine	36.77 ± 14.88	113.20 ± 50.42	318.53 ± 96.98	136.57 ± 41.56	0.06		¥	
Ethanolamine	74.30 ± 10.63	43.65 ± 17.06	141.02 ± 36.73	52.28 ± 17.17	0.06	0.06		
Glycine	180.92 ± 41.47	202.30 ± 103.68	290.37 ± 70.27	116.43 ± 39.40		0.06		
Aspartic Acid	5.03 ± 1.25	2.17 ± 0.74	50.95 ± 23.67	3.82 ± 1.50	0.06	#	¥	
Sarcosine	4.63 ± 1.53	9.13 ± 3.52	17.40 ± 6.47	14.82 ± 4.86				
Citrulline	10.33 ± 2.11	12.07 ± 2.90	25.55 ± 8.33	15.85 ± 3.92				
Glutamic Acid	88.73 ± 24.57	41.97 ± 19.61	302.45 ± 130.13	40.87 ± 13.57	0.06	#		
Beta-Alanine	34.52 ± 12.29	37.40 ± 15.04	67.17 ± 12.05	58.43 ± 12.10				
Threonine	62.08 ± 17.93	63.15 ± 23.52	365.87 ± 125.30	98.80 ± 18.62			¥	
Alanine	62.65 ± 20.35	50.28 ± 15.74	192.63 ± 57.79	109.18 ± 23.47			¥	
Gamma-Amino-N-butyric-acid	12.90 ± 3.70	17.55 ± 7.73	21.83 ± 4.34	16.29 ± 4.84				
Alpha-Aminoadipic-acid	0.28 ± 0.10	0.48 ± 0.21	0.43 ± 0.14	1.05 ± 0.29				
Beta-Aminoisobutyric-acid	3.22 ± 1.06	5.03 ± 1.67	3.56 ± 0.84	3.18 ± 0.89				
Proline	19.07 ± 4.87	15.70 ± 6.99	62.23 ± 15.13	24.65 ± 5.82			¥	
Hydroxylysine 1	0.00 ± 0.00	0.00 ± 0.00	0.00 ± 0.00	0.00 ± 0.00				
Hydroxylysine 2	0.54 ± 0.08	0.66 ± 0.16	0.67 ± 0.06	0.70 ± 0.09				
Alpha-Amino-N-butyric-acid	0.80 ± 0.27	1.57 ± 0.29	1.38 ± 0.31	0.75 ± 0.23				0.06
Ornithine	8.15 ± 3.39	7.22 ± 2.21	13.63 ± 2.40	13.54 ± 3.88				
Cystathionine 1	0.10 ± 0.03	0.00 ± 0.00	0.57 ± 0.08	0.30 ± 0.01	*	#	¥	§
Cystathionine 2	0.00 ± 0.00	0.00 ± 0.00	0.00 ± 0.00	0.00 ± 0.00				
Lysine	60.58 ± 20.99	48.77 ± 15.86	343.20 ± 137.15	73.63 ± 17.95			¥	
Cysteine	2.40 ± 0.38	3.77 ± 1.01	2.50 ± 0.50	2.95 ± 0.74				
Tyrosine	9.57 ± 2.68	9.07 ± 3.93	93.53 ± 42.59	10.88 ± 2.81			¥	
Methionine	12.10 ± 4.07	14.70 ± 7.70	107.57 ± 46.38	20.63 ± 5.80			¥	
Valine	19.92 ± 5.57	8.77 ± 3.15	202.92 ± 95.43	13.23 ± 2.88	0.06	#	¥	
Isoleucine	11.05 ± 2.91	4.60 ± 1.78	122.52 ± 58.76	3.97 ± 1.05	0.06	#	0.06	
Allo-Isoleucine	2.42 ± 1.29	1.30 ± 0.43	3.29 ± 0.38	10.21 ± 4.15				§
Homocysteine	0.47 ± 0.06	0.68 ± 0.05	0.80 ± 0.80	0.00 ± 0.00	*		¥	§
Leucine	22.77 ± 6.08	9.25 ± 3.76	281.95 ± 135.86	13.42 ± 3.37	0.06	#	¥	
Phenylalanine	12.45 ± 3.70	15.00 ± 6.30	88.08 ± 38.56	16.15 ± 4.15			¥	
Tryptophan	4.97 ± 0.96	4.67 ± 1.33	29.93 ± 13.48	3.57 ± 0.88		#		

Pairwise comparisons of cystic fluid were conducted using Mann–Whitney *U* tests (non-parametric data), # *p*-value < 0.05. *N* = 6 rats per group.

* Significantly different between Male and Female SD rats

# Significanly different betweem Male and Female PCK Rats

¥ Significantly different between Male SD and PCK rats

§ Significantly different between Female SD and PCK rats

Compared to both plasma and urine values, 6 amino acids were pointedly different in all fluids. These were Alanine, Cysteine, Ornithine, Citrulline, Sarcosine, and α-Aminoadipic Acid. In total, there were 22 amino acids in cystic fluid that were significantly increased above both plasma and urine values: Histidine, Leucine, Isoleucine, Valine, Methionine, Phenylalanine, Lysine, Arginine, Asparagine, Serine, Aspartic Acid, Glutamic Acid, Glycine, Alanine, Tyrosine, Cysteine, 1-Methylhistidine, Sarcosine, Citrulline, Ornithine, Hydroxylysine 2, and α-Aminoadipic-acid. All amino acid comparison graphs can be found in Figs. S3–S6. Regarding sex variations, there were only 4 amino acids in the cystic fluid: 1-Methylhistidine, 3-Methylhistidine, Ethanolamine, and Taurine that differed between males and females, although Sarcosine was trending towards significance (Fig. S7). In all 5 of these, the circulating amino acid level was also significantly distinct between sexes.

**Fig. 7:**
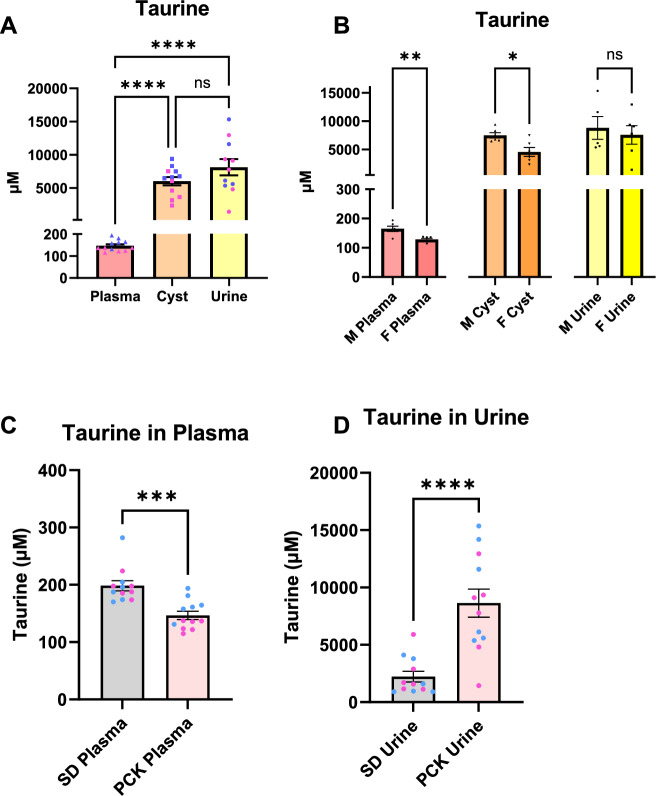
Taurine concentrations. **A** Comparison of taurine concentrations between plasma, cyst fluid, and urine. **B** Comparisons of taurine concentration sex differences. Taurine was significantly reduced in female plasma and cyst fluid. **C** Summary of taurine plasma levels in age-matched Sprague Dawley (SD) rats and PCK rats. **D** Graph of urinary taurine concentrations in SD or PCK rats. There were no significant differences in plasma or urine taurine concentrations in SD rats. Individual male (blue) and female (pink) data points are shown in each graph, mean ± SEM. *N* = 6 male and 6 female rats. Significance was determined by Brown-Forsythe and Welch ANOVA with Dunnet’s correction for multiple comparisons (**A**) or unpaired *t*-test (**B**–**D**) *p < 0.05, **p < 0.01, ***p < 0.001, ***p < 0.0001.

The concentration of taurine in cystic fluid was over 5-fold greater than the next most concentrated amino acid (Fig. 7). Taurine is dramatically increased in the cyst fluid compared to circulating values; however, it was not significantly different from urine. Additionally, circulating and cyst fluid taurine levels were significantly decreased in females. We also measured circulating and urinary taurine levels from male and female SD rats (Tables 1 and 2, respectively). PCK rats had significantly reduced circulating taurine, although they remained within normal circulating taurine values (Fig. 7C). On the other hand, urinary taurine was tremendously increased in the PCK rats compared to SD controls (Fig. 7D). There were no sex differences in SD rat taurine concentrations. It should also be noted that the rat diets contained only 0.00 (PCK)–0.3 (SD)% taurine (Fig. S8). Cysteine dioxygenase (CDO) and cysteine sulfinate decarboxylase (CSAD) are the 2 rate-limiting enzymes responsible for taurine synthesis from cysteine degradation, but the genes encoding these enzymes were not significantly different between male and female PCK rats (data not shown but can be found in RNA-Seq supplementary materials).

## Discussion

In this study, we investigated the ion concentrations and metabolite profiles of cystic fluid from the PCK rat, a model of ARPKD. Our findings demonstrate that cystic fluid in this model has its own distinct ion concentration gradients and that the metabolite profile differs between sexes. Compared to ADPKD distal cyst human values, the rat K^+^ concentrations were much greater (~95 mM vs 5–58 mM), but the Na^+^ (24 vs 5–50 mM) was in the middle of the detected human range, and the Cl- (60 vs 0–55 mM) was just slightly over the maximum reported human values^14^. Ion and solute transport are critically reliant upon unique concentration gradients and membrane voltage potentials. To date, most in vitro transport experiments, including those from our group, have been conducted in cell culture media or extracellular solutions that mimic the interstitial fluid with typical concentrations in the 110–150 mM Na^+^, 3–30 mM K^+^, 1–2 mM Ca^2+^, and 100–140 mM Cl^−^ ranges^36,45–48^. For distal gradient cysts, this solution may not provide accurate information about transport directionality as both ion driving forces and resting apical membrane potential should be impacted by the low Na^+^ and Cl^−^, but high K^+^ extracellular concentrations. Further studies are needed to investigate the potential functional effects this altered apical extracellular solution may have on transport and downstream signaling. Additionally, cyst fluid osmolality is worth noting as a recent preprint from Márquez-Nogueras et al. suggests that polycystin 2 may act as an osmosensor^49^. Interestingly, although we did not observe any significant differences in the cyst fluid ion concentrations, there were significant differences in mRNA expression of several collecting duct transporters that may be protective in females, although this is only speculation without functional channel measurements in males and females. Namely, CFTR and potentially TMEM16A are proposed to be major transporters responsible for chloride secretion into the cysts, which helps drive cystogenesis^41^. The transcripts for both these proteins are significantly reduced in the female PCK kidneys. Additionally, both the α-(*Scnn1a*) and β-(*Scnn1b*) ENaC subunits are significantly increased in females as expected based on previous data in healthy female rodents^42^. The augmented Na^+^ absorption through increased ENaC activity could help prevent fluid buildup in females^44,50^. Furthermore, Arkhipov et al. reported that loss of the ionotropic purinergic receptor P2X7 increased ENaC activity, slowing cyst growth, and the females also have reduced *P2rx7* expression^51^. Whether these transcriptional changes remain at the protein level and translate to functional differences, as well as whether humans with PKD have similar expression profiles, still needs to be determined.

Our results also demonstrated that there is a diverse range of chemical classes present in the cystic fluid, and the predominant chemical class appears to be amino acids, their derivatives, and peptide fragments. Our findings are consistent with previous reports where changes in tryptophan metabolism, arginine biosynthesis, fatty acid metabolism, purine metabolism, folate metabolism, nicotinate/nicotinamide metabolism, and drug metabolism were identified in different animal and human models^23,32,52,53^. Reduction of fatty acid oxidation and dysregulation of lipid metabolism are established features of polycystic kidneys^28^. Previous work from Trott et al. found that arginine deprivation could inhibit cyst growth in vitro and that *Pkd1*-null cell lines had reduced proliferation compared to wild-type cell lines when challenged with reduced arginine levels^27^. Arginine reprogramming affects metabolites in both the urea and tricarboxylic acid (TCA) cycles, and arginine concentration in the PCK cyst fluid was more than double the circulating arginine concentration (Fig. S4). Our observed sex differences in tryptophan metabolism are also noteworthy as they are in line with observations from several other studies. Hopp et al. identified tryptophan metabolites as being highly associated with disease progression in the ADPKD model, the *Pkd1*^RC/RC^ mouse^29^, and building on this foundation, Nguyen et al. demonstrated that knockout of the key tryptophan metabolism enzyme, indoleamine 2,3-dioxygenase 1 (IDO1), in this model reduced cystic index and kidney to body weight ratios^54^. Beyond this, Baliga et al. found that tryptophan metabolites were significantly different between healthy and diseased children and associated with disease progression in a clinical trial of young adults with ADPKD^32^.

Similar to cancer cells, PKD epithelia feature a shift from oxidative phosphorylation to aerobic glycolysis known as the Warburg effect^53,55^. This results in enhanced production of lactate from glycolysis instead of the full utilization of glucose through the TCA cycle. While we did not observe differences in the lactate present between male and female PCK rats, we did find lactate present in the cystic fluid. Also related to this was the high concentration of glucose present in the PCK rat cystic fluid at 70 mg/dL, which could be contributing to the shift to aerobic glycolysis. Indeed, high glucose has been demonstrated to increase cystogenesis in PKD organoids^56^, and increased pancreatic cyst glucose is a diagnostic marker for potentially malignant cysts^57^. Moreover, a recent randomized controlled trial with 66 ADPKD patients utilizing a ketogenic diet showed an improved kidney function in ADPKD patients; however, the kidney size decrease did not reach statistical significance^58^.

Perhaps the most unexpected finding of this study was the high taurine content in the cystic fluid and urine from the PCK rats. However, previous metabolomics studies have associated it with cystic disease in urine. Work from Taylor et al. characterizing the urinary metabolome of the renal cyst model, the *jck* mouse, identified taurine as one of the top 50 metabolites that could discriminate between WT and diseased mice, although in their model, urinary taurine excretion was reduced in *jck* mice^23^. Additionally, taurine and hypotaurine metabolism was identified as a pathway of interest in the joint pathway analysis, revealing both metabolite and gene expression changes (Fig. 5, *on the graph).

Taurine is one of the most abundant amino acids in the body and has many different functions, including regulating cell volume, membrane stabilization, formation of bile salts, and scavenging free radicals^59^. Systemic taurine levels are maintained through the conversion of cysteine to taurine and taurine consumption^59^. Since mammals lack the enzymatic machinery to break down taurine, excess taurine is either excreted by the kidneys or eliminated through the feces as a bile-salt conjugate^59^. Taurine uptake into cells occurs predominantly through the Na^+^ Cl^−^ dependent taurine transporter (TauT, encoded by *Slc6a6*) but can also pass through the higher capacity but lower affinity proton-coupled amino acid transporter, PAT1. Beyond this, some amino acids, such as β-alanine can outcompete taurine for TauT transport, allowing for further reduced systemic taurine levels^59^.

In the kidney, taurine acts as an organic osmolyte to protect medullary cells in hypertonic conditions and has been shown to accumulate in the proximal tubule, thin descending limb, thick ascending limb, and collecting duct cells^60,61^. In most cases, taurine and taurine transport are renoprotective; taurine supplementation reduced blood pressure increases in Dahl Salt-Sensitive rats^62^, prior taurine administration reduced ischemia-reperfusion injury^63^, loss of TauT increased diabetic nephropathy susceptibility in streptozotocin-treated C57BL/6 mice^64^, and taurine transporter knockout mice had reduced diuresis after repeated water loads and increased vasopressin to creatinine ratios following water deprivation^65^. Patients with ESKD have lower circulating taurine, and urinary taurine is reduced in patients with advanced CKD^66,67^. In the case of our model of ARPKD; however, we postulate that it may be deleterious as taurine accumulation in the cyst fluid could provide solute drag to increase cyst growth. While initially potentially beneficial by acting as an antioxidant and preventing cell volume changes, taurine may then get trapped within cysts. Furthermore, the low Na^+^ and Cl^−^ concentrations in the cyst fluid would reduce apical TauT activity, increasing the likelihood that taurine accumulates in the cyst fluid and is unable to reenter the cell due to unfavorable concentration gradients. Future studies will more thoroughly interrogate this potential phenomenon.

From a clinical perspective, the results of this work have several interesting implications for potential dietary intervention or therapeutic targets for PKD patients. A recent study from Kim et al. found that plasma taurine levels of ADPKD patients were inversely correlated with baseline height to total kidney volume, a measurement of cyst burden, suggesting taurine may be important in human PKD^31^. Additionally, in the human ADPKD scRNA-Seq data set from Muto et al., there are notable changes in TauT mRNA expression in several cell types compared to control kidneys, further strengthening the rationale to study taurine transport and metabolism in PKD^68^. Ongoing studies in the lab are assessing taurine levels and untargeted metabolomics of cystic fluid and plasma from ADPKD patients to more thoroughly investigate this translational potential. In humans, our taurine intake comes from animal protein, so a vegan diet has virtually no dietary taurine; normal levels come from endogenous production. If dietary taurine is an environmental factor that exacerbates cystogenesis, clinicians could advise patients to avoid taurine supplements, energy drinks, and animal proteins high in taurine, such as shellfish. Regarding sex differences, a study examining gender-specific pathways in humans found that the cysteine-methionine-S-Adenosylmethionine-taurine pathway was significantly increased in males^69^. Beyond this, taurine is important for male reproductive function, and testosterone has been shown to increase taurine synthesis through elevated CSAD expression in mouse hepatocytes, whereas estradiol had the opposite effect^70–72^. In summary, the results of this study demonstrate that there is still more to learn about the composition of polycystic kidney fluid, and this knowledge may provide insights for potential new pharmacological interventions or dietary changes that can help slow disease progression.

## Methods

### Animals

Rats were maintained in a standard 12/12 dark/light cycle with food provided *ad libitum*. For experiments, 10–12 week-old rats were deeply anesthetized with isoflurane and distal aorta cannulated for arterial blood collection prior to flushing the kidneys with PBS. Urine samples were collected directly from the bladder by aspiration with a 22-gauge needle and 1 mL syringe. All samples were collected between 11 and 3 pm to eliminate potential circadian effects. Diet compositions can be found in Fig. S8. All animal procedures were approved by the Institutional Animal Care and Use Committee at the University of South Florida (USF), Medical University of South Carolina (MUSC) or Medical College of Wisconsin (MCW) in accordance with the Guide for the Care and Use of Laboratory Animals and followed the ARRIVE guidelines. We have complied with all relevant ethical regulations for animal use.

### Untargeted metabolomics and RNAseq datasets

Male and Female PCK rats (PCK/CrljCrl-Pkhd1pck/Crl) were previously purchased from Charles River and a colony was maintained at MCW on Purina Lab Diet 5001. The 5 male and 5 female PCK rats used for untargeted metabolomics were littermates from 2 separate litters with different parents; all samples were collected within 2 weeks of one another and immediately snap-frozen.

### Targeted metabolomics

Male and Female PCK rats were obtained from the PKD Research Resource Consortium (PKD-RRC) and housed at MUSC. Tissue was collected from an active breeding colony at MUSC maintained on PicoLab Verified 75 IF 5V75 lab diet (0.0% Taurine, Fig. S8).

Sprague-Dawley rats were processed at USF. Sprague Dawley rats were obtained from Charles River, maintained on Teklad 2918 irradiated diet (<0.3% Taurine^73^, Fig. S8), and allowed to acclimate to their new location for 2 weeks before tissue collection.

### Biochemical measurements

The concentrations of Na^+^, K^+^, Cl^−^, Ca^2+^, glucose and creatinine from urine, blood, and cyst fluid were measured with a blood gas analyzer ABL800 FLEX (Radiometer America Inc.); samples were analyzed in freshly collected samples using the same instrument. Osmolarity measurements were determined by the MCW Department of Physiology Biochemistry Core Laboratory using freezing point depression. Due to variations in the amount of available cyst fluid and urine, several measurements were missing and group size may vary.

### Untargeted metabolomics

Methodology for Qualitative large-scale profiling as previously published^74,75^. Cyst fluid samples were deproteinized with six times the volume of cold acetonitrile:methanol (1:1 ratio), kept on ice with intermittent vortexing for 30 min at 4 C, then centrifuged at 18000 × *g*. 13C6-phenylalanine (3 µl at 250 ng/µl) was added as an internal standard to each sample prior to deproteinization. The supernatants were divided into 2 aliquots and dried down for analysis on a Quadrupole Time-of-Flight Mass Spectrometer (Agilent Technologies 6550 Q-TOF) coupled with an Ultra High Pressure Liquid Chromatograph (1290 Infinity UHPLC Agilent Technologies). Profiling data were acquired under both positive and negative electrospray ionization conditions over a mass range of 100–1200 m/z at a resolution of 10,000–35,000 (separate runs). Metabolite separation was achieved using two columns of differing polarity, a hydrophilic interaction column (HILIC, ethylene-bridged hybrid 2.1 × 150 mm, 1.7 mm; Waters) and a reversed-phase C18 column (high-strength silica 2.1 × 150 mm, 1.8 mm; Waters). For each column, the run time is 20 min using a flow rate of 400 µl/min. A total of four runs per sample were performed to give maximum coverage of metabolites. Samples were injected in duplicate or triplicate, and a quality control sample, made up of a subset of samples from the study was injected several times during a run. All raw data files obtained were converted to compound exchange file format using Masshunter DA reprocessor software (Agilent). Mass Profiler Professional (Agilent) was used for data alignment and to convert each metabolite feature (m/z × intensity × time) into a matrix of detected peaks for compound identification. An unsupervised principal component analysis (PCA), ANOVA, and heat map comparison between groups were obtained for analysis. This gives a list of accurate mass molecular weights of differentially expressed components that was run against the Metlin database to give putative identification (IDs). The list of components would have a putative ID or a mass (m/z) value depending on whether match was found. Components that were assigned putative IDs were further examined by comparison to a purchased reference standard of the proposed compound. Mass accuracy of the Q-TOF method was ≤ 0.05 and |fold change|≥ 1.5).

### Metaboanalyst 6.0 MS peaks to pathways

In order to improve MS resolution, rather than identifying unique peaks, a key concept is to identify compounds related to individual pathways or groups of functionally related compounds such as metabolite sets^76^. The general assumption is that the collective behavior of a group is more robust against a certain degree of random errors of individuals. The mummichog algorithm is the implementation of this concept to infer pathway activities from a ranked list of MS peaks identified by untargeted metabolomics. The algorithm uses over-representation analysis (ORA) to determine pathway-level enrichment. A pre-determined cutoff based on p-values is determined by the user. For additional detail, refer to Li et al.^77^. As a complement, Gene Set Enrichment Analysis (GSEA) can be used to extract biological meaning from a ranked gene list. This method considers the ranks of MS peaks without a significance cutoff, and can detect subtle and consistent changes which may be missed by ORA. The mummichog algorithm (Version 2.0) and the adapted GSEA method are combined in the MS Peaks to Paths module. A list of metabolites (the same length as the number of significant m/z features) are inferred from the user’s uploaded set of m/z features, considering all potential matches (isotopes/adducts). Then, the potential compounds are mapped onto known metabolic pathways for *Rattus norvegicus*. Next, a hypergeometric *p*-value is calculated. After multiple repetitions to calculate the null distribution of *p*-values for all pathways, this is modeled as a Gamma distribution. Following this, the significant m/z features are used to calculate the p-values for each pathway. Last accession date: 3-11-24, version 6.0.

### Targeted amino acid panel

Amino acids and their metabolites were measured by liquid-chromatography mass spectrometry as previously described^78^. Briefly, 20 µl of plasma, urine or cystic fluid was spiked with an internal standard solution consisting of isotopically labeled amino acids. The supernatant was immediately derivatized with 6-aminoquinolyl-Nhydroxysuccinimidyl carbamate according to Waters’ AccQ-Fluor kit. A 10-point calibration standard curve underwent similar derivatization procedure after the addition of internal standards. Both derivatized standards and samples were analyzed on a Thermo Quantiva triple quadrupole mass spectrometer coupled with a Waters Acquity liquid chromatography system. Data acquisition was done using select ion monitor (SRM) via positive electrospray condition. Concentrations of 42 analytes of each unknown were calculated against its respective calibration curve.

### RNA extraction, RNA-Seq, and data analysis

RNA was isolated from snap-frozen kidney cortex tissue of 10–12 week-old male and female PCK rats using TRIzol according to the manufacturer’s instructions. Sequencing, mapping, and initial analysis was performed by Novogene. After passing quality control, DNA libraries were sequenced by Illumina HiSeq 2500. Genome was mapped using HISAT2 to reference genome: ensembl_rattus_norvegicus_rnor_6_0_gca_000001895_4. For quantification, gene expression levels were estimated from the abundance of transcripts that mapped to the genome or exon using FPKM (Fragments Per Kilobase of transcript sequence per Millions base pairs sequenced). After quantification of gene expression, statistical analysis of differentially expressed genes was performed with DESeq2 software, with differential gene screening threshold |log2(FoldChange)|≥ 1 & padj ≤ 0.05. Major Cellular and Molecular functions were determined using IPA, and additional pathway analyses were performed with clusterProfiler software to determine gene ontology and KEGG pathway enrichment. For comparison of specific genes of interest, FPKM values were graphed and analyzed independently. The creation of a heatmap with hierarchical clustering, Principal Component Analysis (PCA) plots, and volcano plots was done using the open-source software R. To achieve this, several R packages were utilized, including ggplot, ggplot2, dplyr, pheatmap, reactlog, circlize, ggrepel, and tidyverse.

### Statistics and reproducibility

Specific test descriptions and sample sizes are given in the figure legends. The data are displayed as the mean ± the standard error of the mean (SEM). The number of rats (replicates) used for analysis is given in each figure legend. For comparison of 2 groups, unpaired student  t-tests (two-tailed) were applied. For plasma vs. cyst fluid vs. urine comparisons, different fluids had different standard deviations, so Brown-Forsythe and Welch ANOVA with Dunnet’s correction for multiple comparisons was used. A comparison of the targeted amino acid panel was performed with assistance from the USF Research Methodology and Biostatistics Core. Pairwise comparisons of cystic fluid were conducted using Mann–Whitney *U* tests (non-parametric data). Significance in enrichment analyses in Metaboanalyst was calculated with the mummichog algorithm. The original algorithm implements an ORA method to evaluate pathway-level enrichment based on significant features.

## Supplementary information

Supplemental Material

Description of Additional Supplementary File

Supplementary Data

## Data Availability

RNAseq data that support the findings of this study have been deposited in GEO. Accession number: GSE261500 (https://www.ncbi.nlm.nih.gov/geo/query/acc.cgi?acc=GSE261500). The description of numerical and algorithm data used to generate figures is given in the Supplementary Information Fig. S9. Raw untargeted metabolomics data were generated by the Mayo Metabolomics Core Facility, and the processed data may be found in the Supplementary Data (S1–S3). Targeted amino acid panel data is available (S6 andS7). Metaboanalyst mummichog algorithm results used to generate the pathway analyses graphs is in Supplementary Data (S8–S10), and the PCK rat biometrics, ion concentrations, and osmolalities are under S11 in the excel file. Data is separated by individual tabs in the Supplemental Data Excel File.

## Code availability

Metaboanalyst is open-source software used to generate the data in Figs. 2 and 5. Original R code from each specific analysis on the date produced is available upon request. The company, Novogene, provided the results for the RNAseq dataset. This was then verified and re-analyzed and used to make better graphs in R by a blinded co-author, whose code is available upon request.
