# Supplementary material for: Electrolyte and metabolite composition of cystic fluid from a rat model of ARPKD
Source: Commun Biol. 2025 Feb 13;8:230. doi: 10.1038/s42003-025-07631-w (PMC11825955; doi:10.1038/s42003-025-07631-w)
Supplement: Supplementary file 1 — Supplemental Material [file 42003_2025_7631_MOESM1_ESM.pdf]

SUPPLEMENTARY INFORMATION

Electrolyte and Metabolite Composition of Cystic Fluid from a rat model of ARPKD

Christine A. Klemens[1,2,*], Mykhailo Fedoriuk[3], Marharyta Semenikhina[3], Mariia Stefanenko[3], Adrian Zietara[1], Vladislav Levchenko[1], Lashodya V. Dissanayake[1], Oleg Palygin[3], Alexander Staruschenko[1,2,4,*]

[1]Department of Molecular Pharmacology and Physiology, University of South Florida, Tampa, FL 33602

[2]Hypertension and Kidney Research Center, University of South Florida, Tampa, FL 33602

[3]Department of Medicine, Medical University of South Carolina, Charleston, SC 29425

[4]James A. Haley Veterans' Hospital, Tampa, FL 33612

Running title: ARPKD Cystic Fluid Composition

***Correspondence:** cklemens@usf.edu or staruschenko@usf.edu

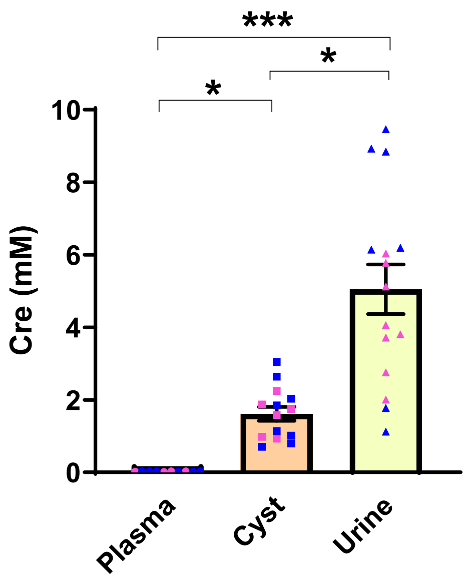

Fig. S1 | Creatinine concentrations. Creatinine levels from plasma, cystic fluid, and urine were measured in both male and female PCK rats. Kruskal-Wallis test with Dunn's correction for multiple comparisons with *p <0.05, ***p <0.001 considered significant. N ≥ 5 male, 5 female rats.

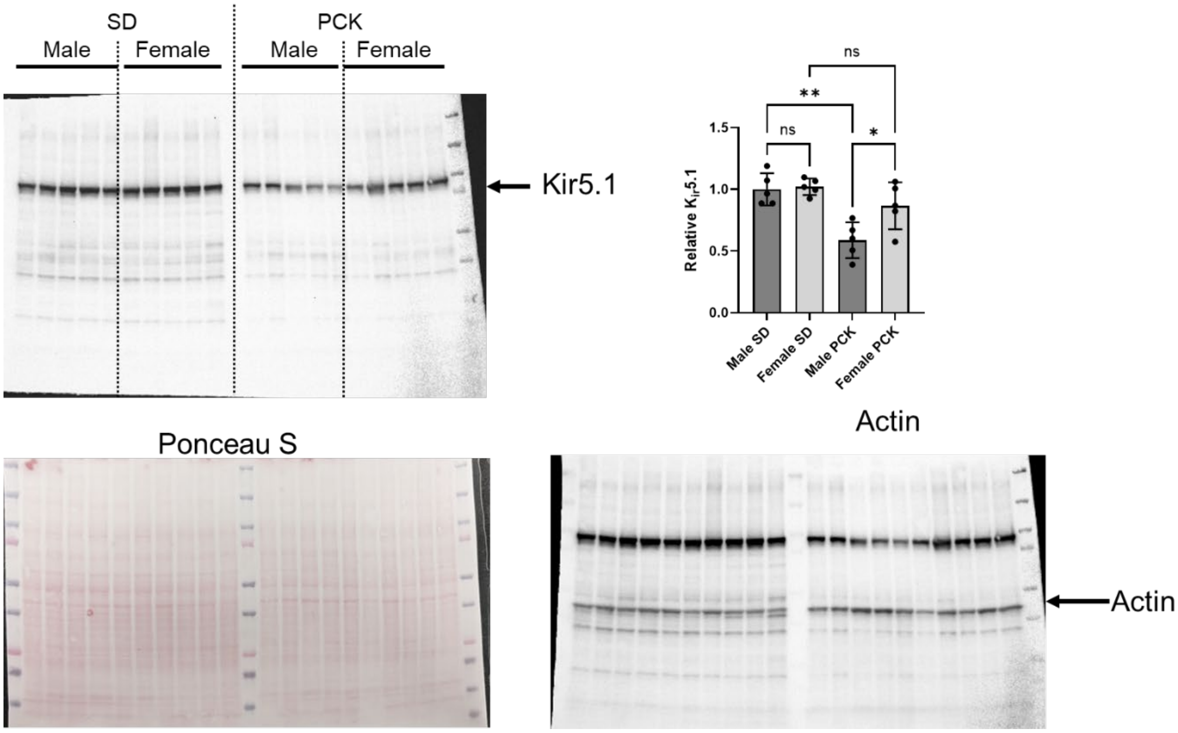

Fig. S2 | Kir5.1 Expression. Western blot of Kir5.1 (*Kcnj16*) from Sprague Dawley (SD) and PCK rats. Both male and female protein levels were assessed. Two-way ANOVA with Holm-Sidak adjustment for multiple comparisons with *p <0.05, **p <0.01 considered significant. N = 5 rats per group; each band is an individual rat.

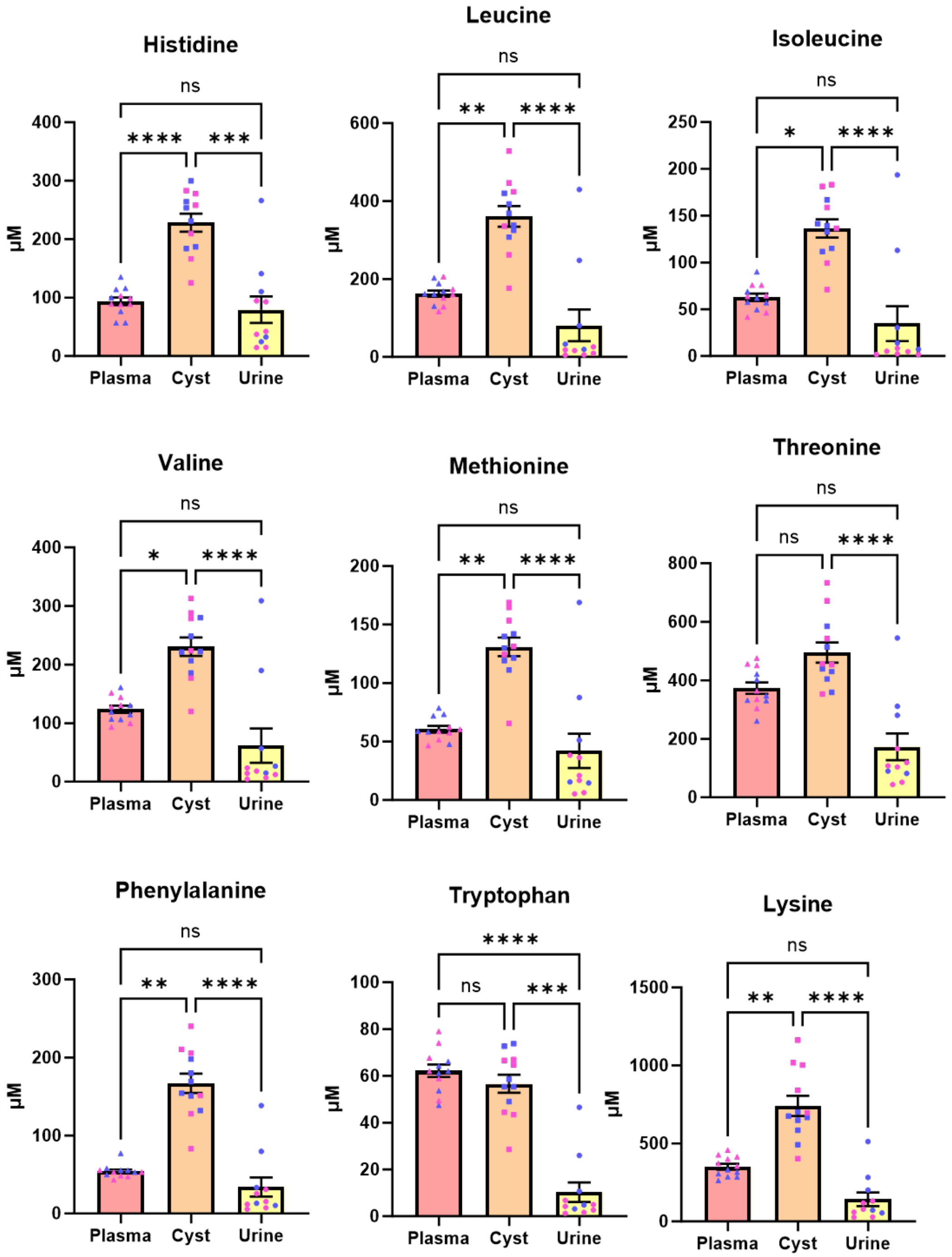

Fig. S3 | Amino acid concentration comparisons for plasma, cyst fluid, and urine. Individual male (blue) and female (pink) data points are shown in each graph. N = 6 male, 6 female rats. Graphs demonstrate the mean ± SEM. Significance considered as *$p<0.05$ **$p<0.01$ ***$p<0.001$. Standard deviations are not the same between fluid groups, so significance was determined by Brown-Forsythe and Welch ANOVA with Dunnet's correction for multiple comparisons or the Kruskal-Wallis test with Dunn's correction for multiple comparisons for datasets that were non-parametric.

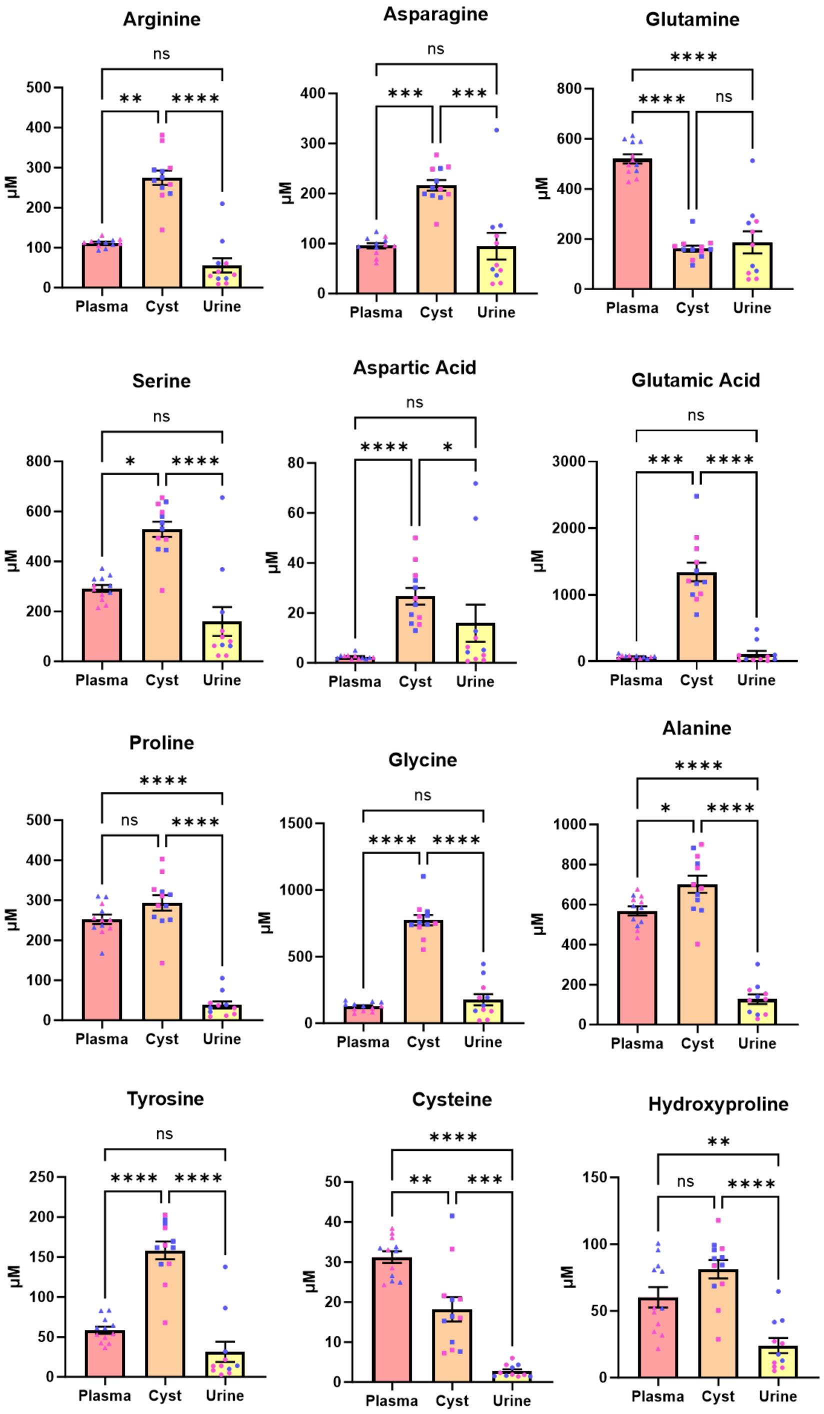

Fig. S4 | Amino acid concentration comparisons for plasma, cyst fluid, and urine. Individual male (blue) and female (pink) data points are shown in each graph. N = 6 male, 6 female rats. Graphs demonstrate the mean ± SEM. Significance considered as *$p<0.05$ **$p<0.01$ ***$p<0.001$. Standard deviations are not the same between fluid groups, so significance was determined by Brown-Forsythe and Welch ANOVA with Dunnet's correction for multiple comparisons or the Kruskal-Wallis test with Dunn's correction for multiple comparisons for datasets that were non-parametric.

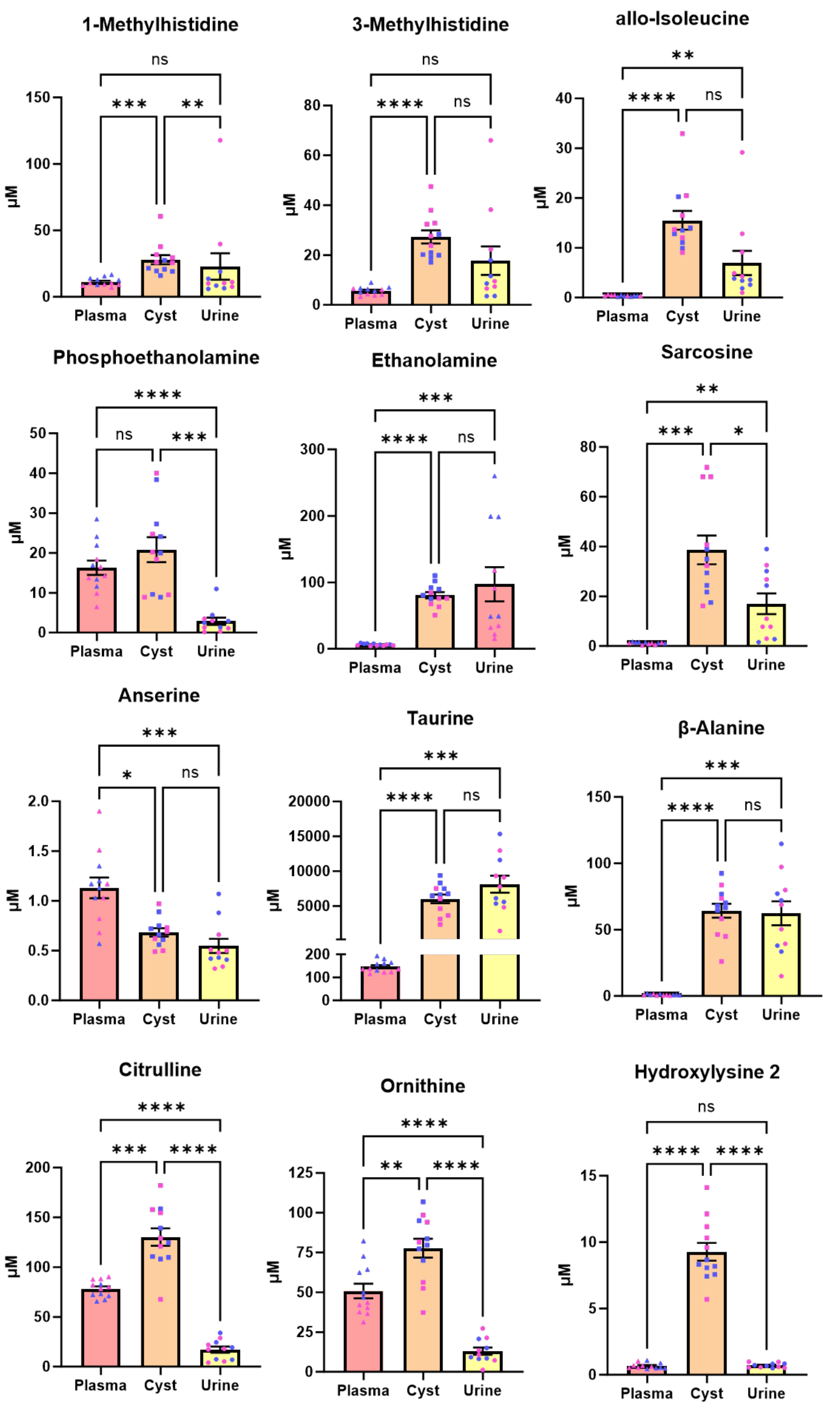

Fig. S5 | Amino acid concentration comparisons for plasma, cyst fluid, and urine. Individual male (blue) and female (pink) data points are shown in each graph. N = 6 male, 6 female rats. Graphs demonstrate the mean ± SEM. Significance considered as *$p<0.05$ **$p<0.01$ ***$p<0.001$. Standard deviations are not the same between fluid groups, so significance was determined by Brown-Forsythe and Welch ANOVA with Dunnet's correction for multiple comparisons or the Kruskal-Wallis test with Dunn's correction for multiple comparisons for datasets that were non-parametric.

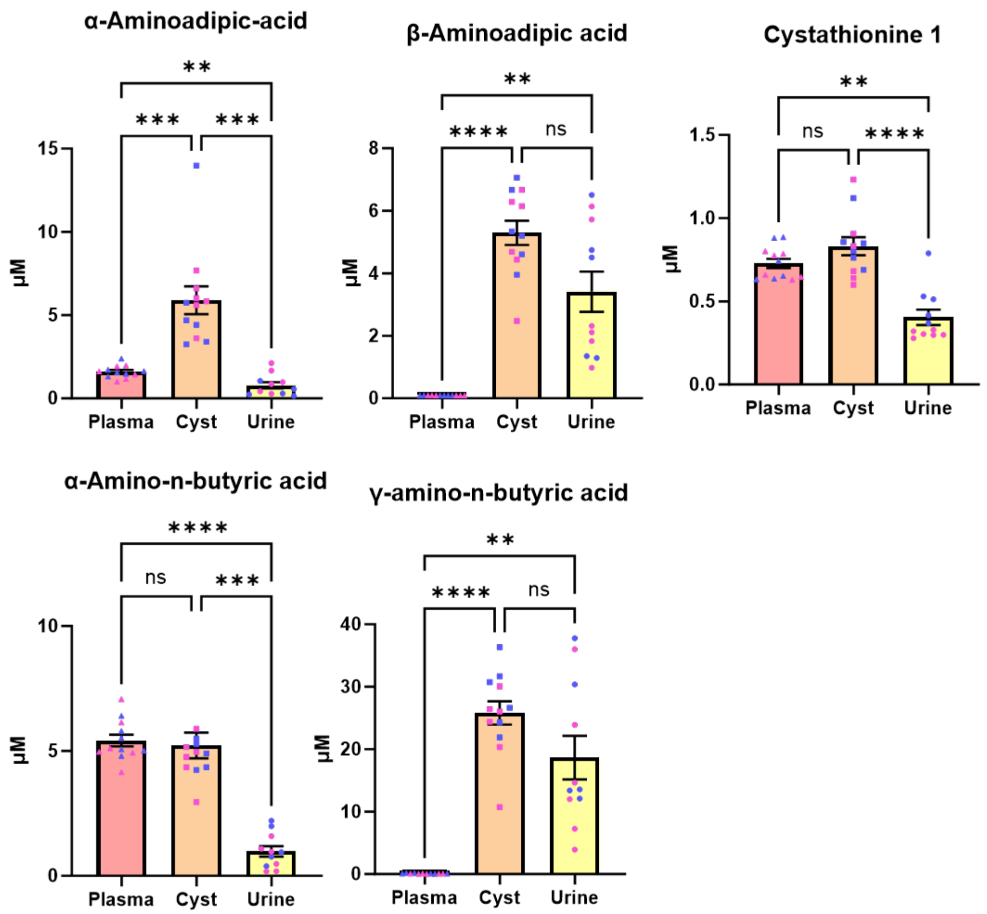

Fig. S6 | Amino acid concentration comparisons for plasma, cyst fluid, and urine. Individual male (blue) and female (pink) data points are shown in each graph. N = 6 male, 6 female rats. Graphs demonstrate the mean ± SEM. Significance considered as *p<0.05 **p<0.01 ***p<0.001. Standard deviations are not the same between fluid groups, so significance was determined by Brown-Forsythe and Welch ANOVA with Dunnet's correction for multiple comparisons or the Kruskal-Wallis test with Dunn's correction for multiple comparisons for datasets that were non-parametric.

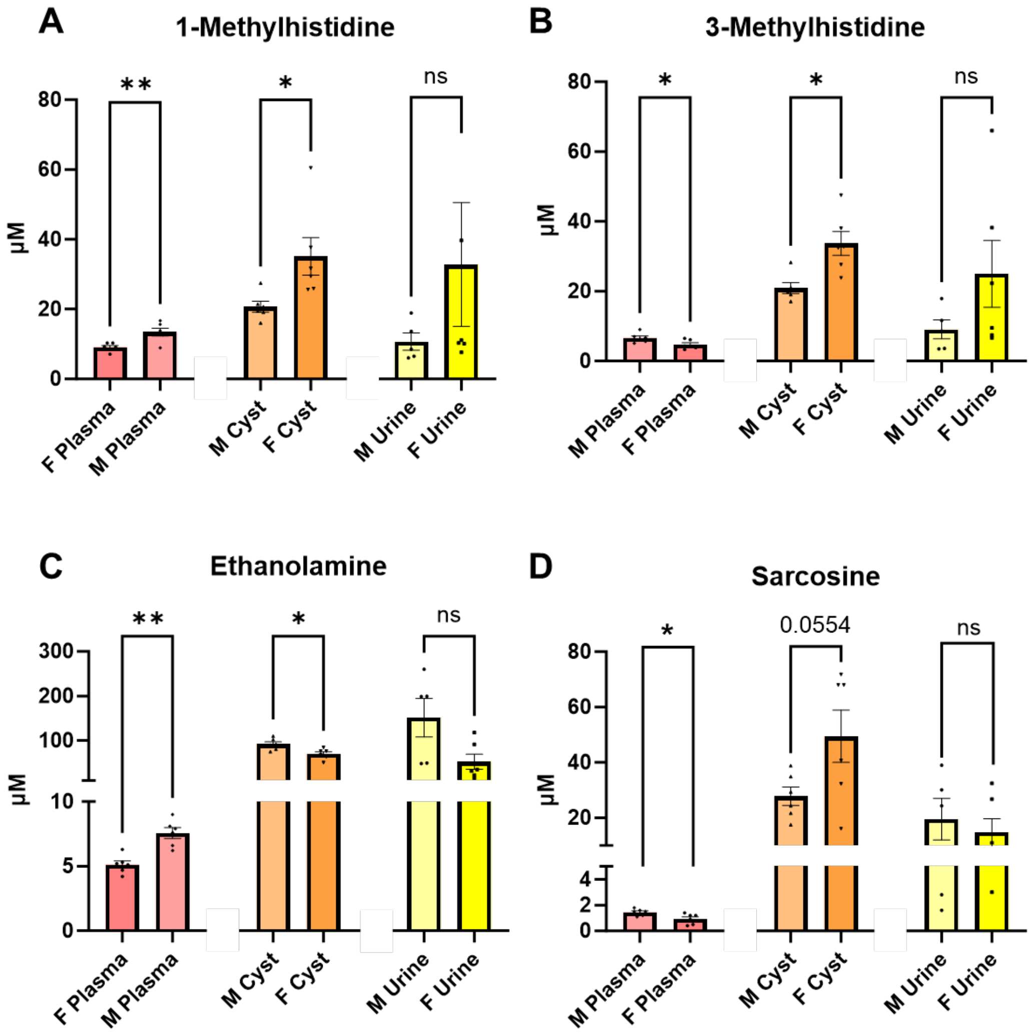

Fig. S7 | Sex differences in plasma, cyst fluid and urine amino acid concentrations. Of the tested amino acids, 4 were significantly different between males (M) and females (F). A) 1-Methylhistidine, B) 3-Methylhistidine, C) Ethanolamine, and Taurine (see Fig. 9). D) Sarcosine trended toward significance. Plasma and urine values for each amino acid are also included for comparison. Graphs demonstrate the mean ± SEM. N = 6 male and 6 female rats. Significance was determined by unpaired t-test with *p<0.05 **p<0.01 ***p<0.001.

Sprague Dawley Diet

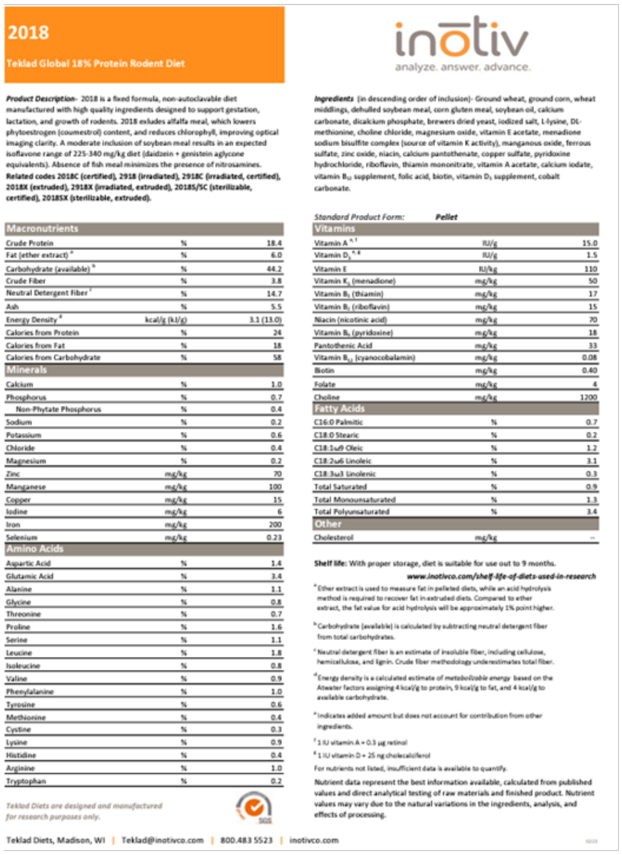

2018

Teklad Global 18% Protein Rodent Diet

Product Description- 2018 is a fixed formula, non-autoclavable diet manufactured with high quality ingredients designed to support gestation, lactation, and growth of rodents. 2018 exludes alfalfa meal, which lowers phytoestrogen (coumestrol) content, and reduces chlorophyll, improving optical imaging clarity. A moderate inclusion of soybean meal results in an expected isoflavone range of 225-340 mg/kg diet (daidzein + genistein aglycone equivalents). Absence of fish meal minimizes the presence of nitrosamines. **Related codes 2018C (certified), 2918 (irradiated), 2918C (irradiated, certified), 2018X (extruded), 2918X (irradiated, extruded), 2018S/SC (sterilizable, certified), 2018SX (sterilizable, extruded).**

Macronutrients		
Crude Protein	%	18.4
Fat (ether extract) [a]	%	6.0
Carbohydrate (available) [b]	%	44.2
Crude Fiber	%	3.8
Neutral Detergent Fiber [c]	%	14.7
Ash	%	5.5
Energy Density [d]	kcal/g (kJ/g)	3.1 (13.0)
Calories from Protein	%	24
Calories from Fat	%	18
Calories from Carbohydrate	%	58
**Minerals**		
Calcium	%	1.0
Phosphorus	%	0.7
Non-Phytate Phosphorus	%	0.4
Sodium	%	0.2
Potassium	%	0.6
Chloride	%	0.4
Magnesium	%	0.2
Zinc	mg/kg	70
Manganese	mg/kg	100
Copper	mg/kg	15
Iodine	mg/kg	6
Iron	mg/kg	200
Selenium	mg/kg	0.23
**Amino Acids**		
Aspartic Acid	%	1.4
Glutamic Acid	%	3.4
Alanine	%	1.1
Glycine	%	0.8
Threonine	%	0.7
Proline	%	1.6
Serine	%	1.1
Leucine	%	1.8
Isoleucine	%	0.8
Valine	%	0.9
Phenylalanine	%	1.0
Tyrosine	%	0.6
Methionine	%	0.4
Cystine	%	0.3
Lysine	%	0.9
Histidine	%	0.4
Arginine	%	1.0
Tryptophan	%	0.2

Teklad Diets are designed and manufactured for research purposes only.

inotiv

analyze. answer. advance.

Ingredients (in descending order of inclusion)- Ground wheat, ground corn, wheat middlings, dehulled soybean meal, corn gluten meal, soybean oil, calcium carbonate, dicalcium phosphate, brewers dried yeast, iodized salt, L-lysine, DL-methionine, choline chloride, magnesium oxide, vitamin E acetate, menadione sodium bisulfite complex (source of vitamin K activity), manganous oxide, ferrous sulfate, zinc oxide, niacin, calcium pantothenate, copper sulfate, pyridoxine hydrochloride, riboflavin, thiamin mononitrate, vitamin A acetate, calcium iodate, vitamin B_{12} supplement, folic acid, biotin, vitamin D_3 supplement, cobalt carbonate.

Standard Product Form: ***Pellet***

Vitamins		
Vitamin A [e, f]	IU/g	15.0
Vitamin D_3 [e, g]	IU/g	1.5
Vitamin E	IU/kg	110
Vitamin K_3 (menadione)	mg/kg	50
Vitamin B_1 (thiamin)	mg/kg	17
Vitamin B_2 (riboflavin)	mg/kg	15
Niacin (nicotinic acid)	mg/kg	70
Vitamin B_6 (pyridoxine)	mg/kg	18
Pantothenic Acid	mg/kg	33
Vitamin B_{12} (cyanocobalamin)	mg/kg	0.08
Biotin	mg/kg	0.40
Folate	mg/kg	4
Choline	mg/kg	1200
**Fatty Acids**		
C16:0 Palmitic	%	0.7
C18:0 Stearic	%	0.2
C18:1ω9 Oleic	%	1.2
C18:2ω6 Linoleic	%	3.1
C18:3ω3 Linolenic	%	0.3
Total Saturated	%	0.9
Total Monounsaturated	%	1.3
Total Polyunsaturated	%	3.4
**Other**		
Cholesterol	mg/kg	--

Shelf life: With proper storage, diet is suitable for use out to 9 months.

www.inotivco.com/shelf-life-of-diets-used-in-research

[a] Ether extract is used to measure fat in pelleted diets, while an acid hydrolysis method is required to recover fat in extruded diets. Compared to ether extract, the fat value for acid hydrolysis will be approximately 1% point higher.

[b] Carbohydrate (available) is calculated by subtracting neutral detergent fiber from total carbohydrates.

[c] Neutral detergent fiber is an estimate of insoluble fiber, including cellulose, hemicellulose, and lignin. Crude fiber methodology underestimates total fiber.

[d] Energy density is a calculated estimate of *metabolizable energy* based on the Atwater factors assigning 4 kcal/g to protein, 9 kcal/g to fat, and 4 kcal/g to available carbohydrate.

[e] Indicates added amount but does not account for contribution from other ingredients.

[f] 1 IU vitamin A = 0.3 µg retinol

[g] 1 IU vitamin D = 25 ng cholecalciferol

For nutrients not listed, insufficient data is available to quantify.

Nutrient data represent the best information available, calculated from published values and direct analytical testing of raw materials and finished product. Nutrient values may vary due to the natural variations in the ingredients, analysis, and effects of processing.

Teklad Diets, Madison, WI | Teklad@inotivco.com | 800.483 5523 | inotivco.com

PCK Rat Diet

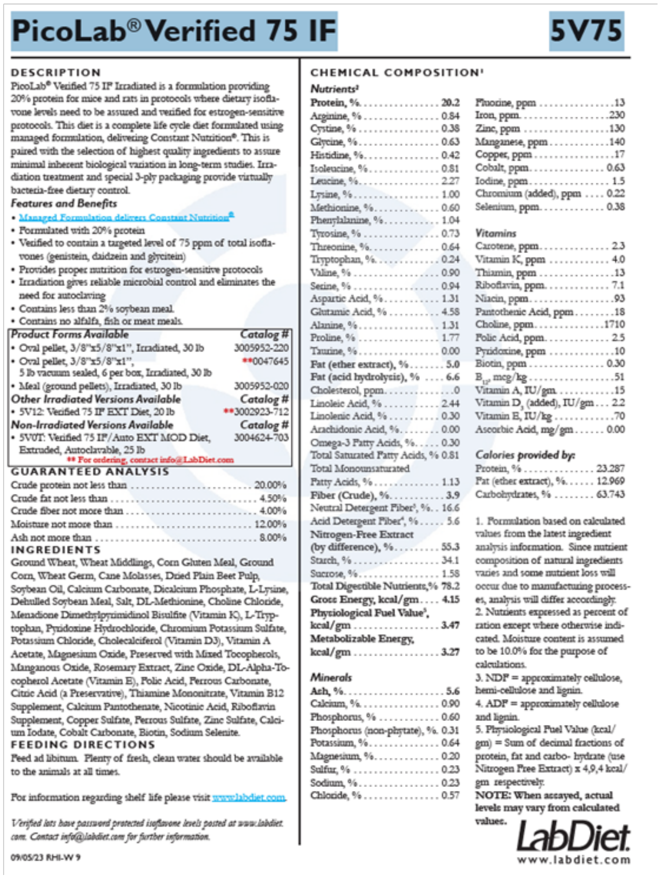

PicoLab® Verified 75 IF **5V75**

DESCRIPTION

PicoLab® Verified 75 IF Irradiated is a formulation providing 20% protein for mice and rats in protocols where dietary isoflavone levels need to be assured and verified for estrogen-sensitive protocols. This diet is a complete life cycle diet formulated using managed formulation, delivering Constant Nutrition®. This is paired with the selection of highest quality ingredients to assure minimal inherent biological variation in long-term studies. Irradiation treatment and special 3-ply packaging provide virtually bacteria-free dietary control.

Features and Benefits

- Managed Formulation delivers Constant Nutrition®
- Formulated with 20% protein
- Verified to contain a targeted level of 75 ppm of total isoflavones (genistein, daidzein and glycitein)
- Provides proper nutrition for estrogen-sensitive protocols
- Irradiation gives reliable microbial control and eliminates the need for autoclaving
- Contains less than 2% soybean meal.
- Contains no alfalfa, fish or meat meals.

Product Forms Available	*Catalog #*
• Oval pellet, 3/8"x5/8"x1", Irradiated, 30 lb	3005952-220
• Oval pellet, 3/8"x5/8"x1", 5 lb vacuum sealed, 6 per box, Irradiated, 30 lb	**0047645
• Meal (ground pellets), Irradiated, 30 lb	3005952-020
Other Irradiated Versions Available	***Catalog #***
• 5V12: Verified 75 IF EXT Diet, 20 lb	**3002923-712
Non-Irradiated Versions Available	***Catalog #***
• 5V0T: Verified 75 IF/Auto EXT MOD Diet, Extruded, Autoclavable, 25 lb	3004624-703

** For ordering, contact info@LabDiet.com

GUARANTEED ANALYSIS

Crude protein not less than	20.00%
Crude fat not less than	4.50%
Crude fiber not more than	4.00%
Moisture not more than	12.00%
Ash not more than	8.00%

INGREDIENTS

Ground Wheat, Wheat Middlings, Corn Gluten Meal, Ground Corn, Wheat Germ, Cane Molasses, Dried Plain Beet Pulp, Soybean Oil, Calcium Carbonate, Dicalcium Phosphate, L-Lysine, Dehulled Soybean Meal, Salt, DL-Methionine, Choline Chloride, Menadione Dimethylpyrimidinol Bisulfite (Vitamin K), L-Tryptophan, Pyridoxine Hydrochloride, Chromium Potassium Sulfate, Potassium Chloride, Cholecalciferol (Vitamin D3), Vitamin A Acetate, Magnesium Oxide, Preserved with Mixed Tocopherols, Manganous Oxide, Rosemary Extract, Zinc Oxide, DL-Alpha-Tocopherol Acetate (Vitamin E), Folic Acid, Ferrous Carbonate, Citric Acid (a Preservative), Thiamine Mononitrate, Vitamin B12 Supplement, Calcium Pantothenate, Nicotinic Acid, Riboflavin Supplement, Copper Sulfate, Ferrous Sulfate, Zinc Sulfate, Calcium Iodate, Cobalt Carbonate, Biotin, Sodium Selenite.

FEEDING DIRECTIONS

Feed ad libitum. Plenty of fresh, clean water should be available to the animals at all times.

For information regarding shelf life please visit www.labdiet.com.

Verified lots have password protected isoflavone levels posted at www.labdiet.com. Contact info@labdiet.com for further information.

09/05/23 RHI-W 9

CHEMICAL COMPOSITION[1]

Nutrients[2]

**Protein, %**	**20.2**
Arginine, %	0.84
Cystine, %	0.38
Glycine, %	0.63
Histidine, %	0.42
Isoleucine, %	0.81
Leucine, %	2.27
Lysine, %	1.00
Methionine, %	0.60
Phenylalanine, %	1.04
Tyrosine, %	0.73
Threonine, %	0.64
Tryptophan, %	0.24
Valine, %	0.90
Serine, %	0.94
Aspartic Acid, %	1.31
Glutamic Acid, %	4.58
Alanine, %	1.31
Proline, %	1.77
Taurine, %	0.00
**Fat (ether extract), %**	**5.0**
**Fat (acid hydrolysis), %**	**6.6**
Cholesterol, ppm	0
Linoleic Acid, %	2.44
Linolenic Acid, %	0.30
Arachidonic Acid, %	0.00
Omega-3 Fatty Acids, %	0.30
Total Saturated Fatty Acids, %	0.81
Total Monounsaturated Fatty Acids, %	1.13
**Fiber (Crude), %**	**3.9**
Neutral Detergent Fiber[3], %	16.6
Acid Detergent Fiber[4], %	5.6
**Nitrogen-Free Extract (by difference), %**	**55.3**
Starch, %	34.1
Sucrose, %	1.58
**Total Digestible Nutrients,%**	**78.2**
**Gross Energy, kcal/gm**	**4.15**
**Physiological Fuel Value[5], kcal/gm**	**3.47**
**Metabolizable Energy, kcal/gm**	**3.27**

Minerals

**Ash, %**	**5.6**
Calcium, %	0.90
Phosphorus, %	0.60
Phosphorus (non-phytate), %	0.31
Potassium, %	0.64
Magnesium, %	0.20
Sulfur, %	0.23
Sodium, %	0.23
Chloride, %	0.57
Fluorine, ppm	13
Iron, ppm	230
Zinc, ppm	130
Manganese, ppm	140
Copper, ppm	17
Cobalt, ppm	0.63
Iodine, ppm	1.5
Chromium (added), ppm	0.22
Selenium, ppm	0.38

Vitamins

Carotene, ppm	2.3
Vitamin K, ppm	4.0
Thiamin, ppm	13
Riboflavin, ppm	7.1
Niacin, ppm	93
Pantothenic Acid, ppm	18
Choline, ppm	1710
Folic Acid, ppm	2.5
Pyridoxine, ppm	10
Biotin, ppm	0.30
B_{12}, mcg/kg	51
Vitamin A, IU/gm	15
Vitamin D_3 (added), IU/gm	2.2
Vitamin E, IU/kg	70
Ascorbic Acid, mg/gm	0.00

Calories provided by:

Protein, %	23.287
Fat (ether extract), %	12.969
Carbohydrates, %	63.743

1. Formulation based on calculated values from the latest ingredient analysis information. Since nutrient composition of natural ingredients varies and some nutrient loss will occur due to manufacturing processes, analysis will differ accordingly.
2. Nutrients expressed as percent of ration except where otherwise indicated. Moisture content is assumed to be 10.0% for the purpose of calculations.
3. NDF = approximately cellulose, hemi-cellulose and lignin.
4. ADF = approximately cellulose and lignin.
5. Physiological Fuel Value (kcal/gm) = Sum of decimal fractions of protein, fat and carbo- hydrate (use Nitrogen Free Extract) x 4,9,4 kcal/gm respectively.

NOTE: When assayed, actual levels may vary from calculated values.

LabDiet.

www.labdiet.com

Fig. S8 | Rodent Diet Formulations from LabDiet and Teklad. Previous work from Singh et al. measured the taurine content of the Teklad 2918 diet and reported it as <0.3% Taurine [1].

1. Singh P*, et al.* Taurine deficiency as a driver of aging. *Science* **380**, eabn9257 (2023).

Fig. S9 – All numerical source data can be found in the Supplementary Data excel file under individual tabs. The description of the data found on each named tab (S1-S11) is given below.

Supplementary Data Excel File: Supplementary Data_CystFluidStudy.xls

S1: Mayo Metabolomics Core Untargeted Metabolomic Analysis

S2: Sample Key for Untargeted Metabolomics

S3: Metaboanalyst Pathway Enrichment Data Input File

S4: Novogene RNAseq Results

S5: Sample Key for RNAseq

S6: Mayo Metabolomics Core Amino Acid Panel – PCK rats

S7: Mayo Metabolomics Core Amino Acid Panel – Sprague Dawley

S8: Metaboanalyst Mummichog Enrichment Results

S9: Metaboanalyst Joint Pathway Analysis Results

S10: Metaboanalyst Joint Pathway Analysis Genes and Compound IDs

S11: PCK Rat 2Kidney:BodyWeight, Ion concentrations, Osmolalities
