# Supplementary material for: Electrolyte and metabolite composition of cystic fluid from a rat model of ARPKD
Source: Commun Biol. 2025 Feb 13;8:230. doi: 10.1038/s42003-025-07631-w (PMC11825955; doi:10.1038/s42003-025-07631-w)
Supplement: Supplementary file 2 — Description of Additional Supplementary File [file 42003_2025_7631_MOESM2_ESM.pdf]

Description Of Additional Supplementary File

File name: Supplementary Data

Description: Numerical and algorithm results used to generate the figures
